# PFKFB4 interacts with FBXO28 to promote HIF-1α signaling in glioblastoma

**DOI:** 10.1038/s41389-022-00433-3

**Published:** 2022-09-17

**Authors:** Emma Phillips, Jörg Balss, Frederic Bethke, Stefan Pusch, Stefan Christen, Thomas Hielscher, Martina Schnölzer, Michael N. C. Fletcher, Antje Habel, Claudia Tessmer, Lisa-Marie Brenner, Mona Göttmann, David Capper, Christel Herold-Mende, Andreas von Deimling, Sarah-Maria Fendt, Violaine Goidts

**Affiliations:** 1 Junior Research Group “Brain Tumor Translational Targets”, German Cancer Research Center (DKFZ), Heidelberg, Germany; 2 Department of Neuropathology, Institute of Pathology, Ruprecht-Karls-University Heidelberg, Im Neuenheimer Feld 224, Heidelberg, Germany; 3 German Consortium of Translational Cancer Research (DKTK), Clinical Cooperation Unit Neuropathology, German Cancer Research Center (DKFZ), Heidelberg, Germany; 4 Laboratory of Cellular Metabolism and Metabolic Regulation, VIB Center for Cancer Biology, VIB, Herestraat 49, 3000 Leuven, Belgium; 5 Laboratory of Cellular Metabolism and Metabolic Regulation, Department of Oncology, KU Leuven and Leuven Cancer Institute (LKI), Herestraat 49, 3000 Leuven, Belgium; 6 Department of Biostatistics, German Cancer Research Center, Heidelberg, Germany; 7 Functional Proteome Analysis, German Cancer Research Center (DKFZ), Heidelberg, Germany; 8 Department of Structural and Functional Genomics, German Cancer Research Center (DKFZ), Heidelberg, Germany; 9 Antibody Unit, Genomics and Proteomics Core Facility, German Cancer Research Center (DKFZ), Heidelberg, Germany; 10 Department of Neuropathology, Charité Universitätsmedizin, Corporate Member of Freie Universität Berlin, Humboldt Universität zu Berlin, Berlin Institute of Health, Berlin, Germany; 11 Division of Neurosurgical Research, Department of Neurosurgery, University Hospital Heidelberg, INF 400, 69120 Heidelberg, Germany

**Keywords:** CNS cancer, Ubiquitylation

## Abstract

Glioblastoma is a highly aggressive brain tumor for which there is no cure. The metabolic enzyme 6-Phosphofructo-2-Kinase/Fructose-2,6-Biphosphatase 4 (PFKFB4) is essential for glioblastoma stem-like cell (GSC) survival but its mode of action is unclear. Understanding the role of PFKFB4 in tumor cell survival could allow it to be leveraged in a cancer therapy. Here, we show the importance of PFKFB4 for glioblastoma growth in vivo in an orthotopic patient derived mouse model. In an evaluation of patient tumor samples of different cancer entities, PFKFB4 protein was found to be overexpressed in prostate, lung, colon, mammary and squamous cell carcinoma, with expression level correlating with tumor grade. Gene expression profiling in *PFKFB4*-silenced GSCs revealed a downregulation of hypoxia related genes and Western blot analysis confirmed a dramatic reduction of HIF (hypoxia inducible factor) protein levels. Through mass spectrometric analysis of immunoprecipitated PFKFB4, we identified the ubiquitin E3 ligase, F-box only protein 28 (FBXO28), as a new interaction partner of PFKFB4. We show that PFKFB4 regulates the ubiquitylation and subsequent proteasomal degradation of HIF-1α, which is mediated by the ubiquitin ligase activity of FBXO28. This newly discovered function of PFKFB4, coupled with its cancer specificity, provides a new strategy for inhibiting HIF-1α in cancer cells.

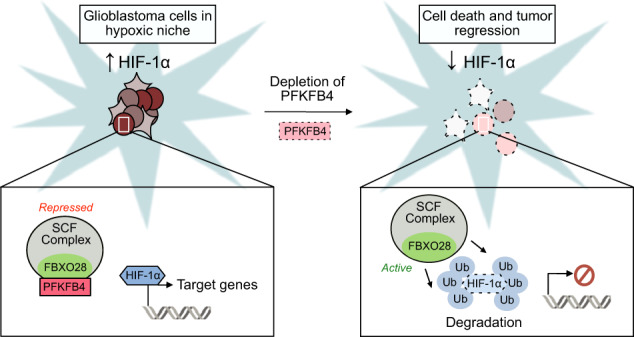

## Introduction

Glioblastoma is one of the most aggressive primary brain tumors in adults, with a dismal median overall survival of only 15 months after diagnosis [1, 2]. As is the case for many tumor types, glioblastoma comprises a highly plastic cellular differentiation hierarchy, with stem-like cells at the apex [3–5]. Thought to be major contributors to patient relapse, these glioblastoma stem-like cells (GSCs) are particularly resistant to chemo- and radiotherapy [6, 7] and are capable of self-renewal and tumor initiation [4, 8]. Therefore targeting this sub-population of glioblastoma cells could lead to more effective therapies for this deadly disease.

Metabolic reprogramming, including upregulation of glycolysis, is one mechanism by which cancers enable rapid cellular proliferation under adverse conditions. PFKFB4 is one of the four isoforms of the key glycolytic regulator 6-phosphofructo-2-kinase/fructose 2,6-bisphosphatase (PFK-2/FBPase-2), involved in the conversion of fructose-6-phosphate (F6P) to fructose 1,6 bisphosphate (FBP) by phosphofructokinase 1 (PFK1). The PFK-2/FBPase-2 isoforms vary in their tissue expression and kinase:phosphatase activity. Both PFKFB3 and PFKFB4 have been shown to be overexpressed in cancer [9–14]. However, of late, PFKFB4 has been gaining interest in its own right. Of particular relevance to glioblastoma is the finding that *PFKFB3:PFKFB4* mRNA ratios are of prognostic value to high grade glioma, with increasing *PFKFB4* levels correlating unfavorably with patient survival [15]. PFKFB4 has also been implicated in cell survival in prostate cancer by regulating antioxidant production through the pentose phosphate pathway (PPP) [16], and PFKFB4 has also been shown to be associated with PPP activity in clear-cell renal cell carcinoma [14]. Furthermore, a new function for PFKFB4 in the activation of steroid receptor coactivator 3 (SRC-3), a transcriptional coactivator, has recently been shown in breast cancer [17]. In a previous study, we have shown that PFKFB4 is upregulated in GSCs compared to normal brain, and that its silencing leads to apoptotic cell death in GSCs [18], highlighting its potential as a therapeutic target in glioblastoma. However, the mechanism of PFKFB4 in GSC survival is not clear.

Non-physiological levels of oxygen tension, or hypoxia, is a common feature of many human cancers. Hypoxia inducible factors (HIFs) are frequently upregulated in cancer as an adaptive mechanism for cells to regulate expression of genes to enable them to cope with reduced oxygen availability. In particular, hypoxia inducible factor 1-alpha (HIF-1α) levels are elevated in glioblastoma [19], with frequent gene alterations, such as epidermal growth factor receptor (EGFR) amplification, loss of phosphatase and tensin homolog (PTEN) and loss of p53, known to play a role in the upregulation of the protein [20, 21]. A wealth of preclinical data has shown that inhibiting HIFs will improve outcome for cancer patients, with glioblastoma being no exception [22, 23]. Interestingly, a recent study has provided evidence that downregulating HIF-1α in glioblastoma could improve temozolomide response [24], suggesting that inhibiting HIF-1α could be a valuable addition to the current standard treatment for the disease. However, the importance of HIF signaling for oxygen homeostasis in normal tissue means that currently available pan-HIF inhibitors have dose-limiting side effects that may prevent their use as chemotherapeutic agents [25].

In this study, we further evaluated the role of PFKFB4 in the survival of cancer cells, particularly focusing on GSCs. We found a new role for PFKFB4 in the regulation of HIF-1α by derepression of a ubiquitin E3 ligase, FBXO28. This mechanism provides a novel strategy for targeting HIF-1α in a cancer specific manner.

## Results

### PFKFB4 silencing reduces tumor size in vivo

**Fig. 1:**
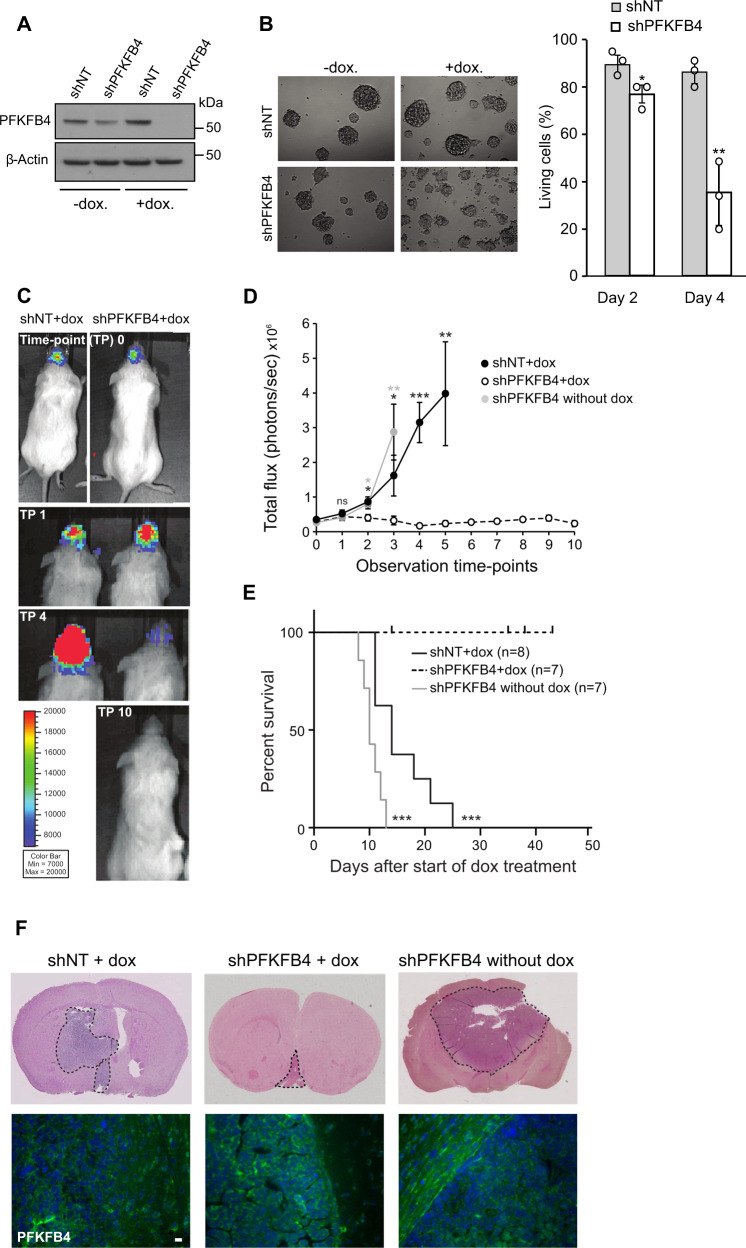
*PFKFB4* silencing reduces tumor size in vivo. **A** PFKFB4 protein levels in luciferase expressing NCH421k GSCs stably transduced with doxycycline inducible shNT or shPFKFB4 on day 4 with or without doxycycline. α-tubulin was used as a protein loading control. **B** (Left) Representative images of NCH421k spheres 6 days after induction of shNT or shPFKFB4 with doxycycline. (Right) Percentage of living cells as determined by propidium iodide (PI) staining and FACS analysis 2 and 4 days after induction of shNT or shPFKFB4 with doxycycline (*n* = 3, mean ± SD, two sided *t*-test, *p value < 0.05, **p value< 0.01). **C** Bioluminescence signal of representative mice orthotopically transplanted with shNT and shPFKFB4 transduced cells at observation time-points 0, 1, 2 and 10, corresponding to 0, 4, 14 and 42 days, after starting administration of doxycycline. **D** Mean bioluminescence signal of mice transplanted with shNT transduced cells (black) and shPFKFB4 transduced cells (black dotted) after start of administration of doxycycline and mice transplanted with shPFKFB4 transduced cells which did not receive doxycycline (gray) at 10 different time points over a period of 35 days. Data are represented as mean ± standard error of the mean (SEM), two-sided *t-*test, *p value < 0.05, **p value < 0.01, ***p value < 0.001. **E** Survival of mice transplanted with shNT (black, *n* = 8) and shPFKFB4 transduced cells (black dotted, *n* = 7) after start of administration of doxycycline, and mice transplanted with shPFKFB4 transduced cells which did not receive doxycycline (gray, *n* = 7). Censored observations are represented by a vertical dash and represent mice which were sacrificed before they displayed any neuropathological symptoms for monitoring tumor growth by immunohistochemistry. Log rank test, ***p value < 0.001. **F** Representative H&E stained tissue sections of brains of a mouse from each group and PFKFB4 protein expression (below). Scale bar = 20 µm.

In order to investigate the importance of PFKFB4 for glioblastoma growth in vivo we used an orthotopic xenograft mouse model. For this purpose, we generated inducible expression constructs encoding shRNA targeting *PFKFB4* and non-target shRNA as a negative control. Patient derived GSC line NCH421k, cultivated as neurospheres in serum-free medium, was stably transduced with the constructs and subsequently treated for 4 days with doxycycline. Using a newly developed antibody (Fig. S1), the knockdown of PFKFB4 was characterized by Western blot (Fig. 1A), and induction of cell death upon knockdown was observed (Fig. 1B). In order to allow the monitoring of the tumor growth in vivo, the inducible GSC lines were stably transduced with a luciferase-expressing construct (Fig. S2A, B). Two groups of 8 and 14 animals were orthotopically transplanted with shNT and shPFKFB4 transduced cells, respectively. When the tumors reached a certain size (around 200,000 flux/photon/second), doxycycline was administered and hence the expression of the shRNA constructs induced (Fig. 1C). Half of the animals with shPFKFB4 containing tumors did not receive doxycycline. Tumor growth was monitored until mice began to show neuropathological symptoms, at which point they were sacrificed. All animals which did not receive doxycycline (7 animals) were sacrificed within 13 days after appearance of the tumor, whereas shNT expressing animals were sacrificed within 25 days, which may suggest a tumor protective effect of doxycycline [26]. Notably, none of the animals whose tumor expressed shPFKFB4 developed symptoms during the follow up (43 days) after appearance of the tumor, and luminescence measurements showed a reduction in tumor size over time, ultimately leading to the complete disappearance of bioluminescence signal in 4 out of 7 mice (Fig. 1D, E). *Post mortem* haemotoxylin and eosin (H&E) staining of the brains of the non-treated shPFKFB4 and the doxycycline treated shNT animals revealed large tumors, while the shPFKFB4 doxycycline-treated mice were tumor-free, or contained only very small tumors. PFKFB4 protein was still present in the residual tumors of the shPFKFB4 expressing animals (Fig. 1F), suggesting that some cells may have escaped PFKFB4 repression in these tumors. In summary, this shows that *PFKFB4* plays a critical role in glioblastoma, its silencing not only slowing tumor growth but resulting in an almost complete eradication of the tumor in this xenograft model.

### Expression of PFKFB4 is cancer specific

Our newly developed antibody is highly specific to PFKFB4, rendering it suitable for immunohistochemistry and allowing the high-throughput staining of tissue microarrays (TMAs). To address the relevance of PFKFB4 in a broader cancer context, we investigated the expression of PFKFB4 protein in patient samples of a range of cancer entities. We found that prostate tumor protein samples expressed higher levels of PFKFB4 than normal samples. Similarly, tumor tissue from lung cancer patients showed elevated protein expression of PFKFB4 compared with matched normal control tissue (Fig. S3A, B). In TMA staining, the level of PFKFB4 protein expression in colon, mammary and squamous cell carcinoma ranges from not expressed (score 0) to highly expressed (score 2), with normal tissue being mostly negative for PFKFB4 (Fig. S3C). Interestingly, the level of expression tends to increase with the grade of the different tumors, irrespective of their tissue origin. This correlation supports PFKFB4 being a relevant therapeutic target, not just in glioblastoma, but also in a range of other cancers.

### PFKFB4 regulates HIF-1α

**Fig. 2:**
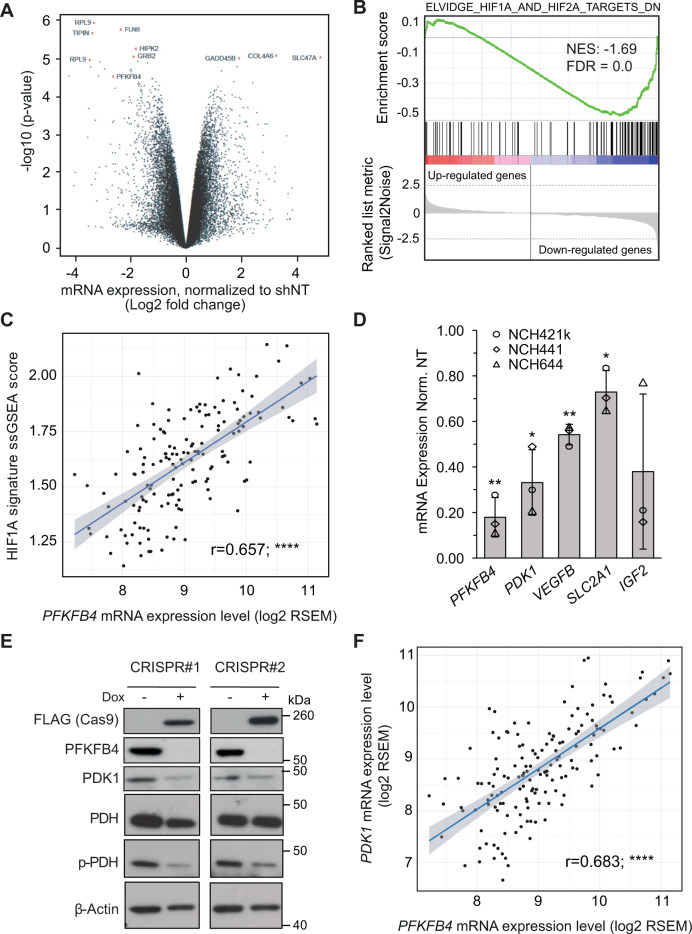
PFKFB4 regulates the transcription factor HIF-1α. **A** Volcano plot showing the fold changes and *p*-values of gene expression in *PFKFB4-*silenced (3 days) GSCs. Data represent mean of the three different patient-derived GSC lines: NCH421k, NCH644 and NCH441. Along with *PFKFB4* itself, the top 10 deregulated probes are highlighted in red and labelled with the gene to which they map. **B** Gene Set Enrichment Analysis (GSEA) comparing a gene signature of downregulated genes in a *HIF1A*/*HIF2A*-silenced breast cancer cell line (from ref. [27], Elvidge) to the gene expression list of *PFKFB4*-silenced GSCs in a rank order based on the mean linear fold change of the genes. The green curve corresponds to the running sum of the enrichment score which reflects the degree to which the gene signature is overrepresented at the bottom of the list. (Normalized enrichment score (NES) = −1.69, false discovery rate (FDR) = 0.0). **C** Correlation between *PFKFB4* mRNA expression and expression of the Elvidge *HIF1A* gene signature based on its single sample gene set enrichment analysis (ssGSEA) score in TCGA glioblastoma patients (regression analysis, *r* = 0.657, ****p value < 0.0001). **D** mRNA levels of *PFKFB4* and selected downregulated HIF-1α target genes (*PDK1, VEGFB, SLC2A1* and *IGF2*) determined by gene expression profiling in three different GSC lines. **E** Protein levels of FLAG (CAS9), PFKFB4, PDK1, PDH and phosphorylated PDH (S293) after doxycycline inducible knockout of *PFKFB4* (7 days incubation with dox). β-actin was used as a loading control. **F** Correlation between *PFKFB4* and *PDK1* mRNA expression in TCGA glioblastoma patients (regression analysis, *r* = 0.683, ****p value < 0.0001).

In order to gain more insight into the role of PFKFB4 in GSC survival, we measured the effect of *PFKFB4* silencing on global gene expression in three different GSC lines (NCH421k, NCH441 and NCH644). A volcano plot of the results is shown in Fig. 2A, with the 10 most deregulated genes labelled. Interestingly, although *PFKFB4* silencing has been shown to reduce expression of SRC-3 target genes transketolase *(TKT)*, adenosine monophosphate deaminase *(AMPD1)* and xanthine dehydrogenase *(XDH)* in breast cancer cells [17], the expression of these genes were not affected in the GSCs (Fig. S4A), suggesting that the mechanism of PFKFB4 in GSC survival is likely to be independent of SRC-3. Gene set enrichment analysis (GSEA) using a collection of curated signatures that represent genetic and chemical perturbations revealed two gene sets involving hypoxia in the top ten downregulated gene sets upon *PFKFB4* silencing [27, 28] (Figs. 2B and S4B, Table S1). The genes in these ten signatures overlapped with each other by less than 19% (Fig. S4C). Expression analysis of the Elvidge HIF-1α and HIF-2α signature in a cohort of glioblastoma patients (*n* = 152), available from The Cancer Genome Atlas (TCGA) [29], revealed a positive correlation between *PFKFB4* expression (*r* = 0.657) and HIF signaling (Fig. 2C). Among the numerous known HIF targets identified to be downregulated upon *PFKFB4* knockdown in GSCs, such as vascular endothelial growth factor B (*VEGFB*), solute carrier family 2 member 1 (*SLC2A1*) and insulin like growth factor 2 (*IGF2*), pyruvate dehydrogenase kinase 1 (*PDK1*) showed the strongest reduction (Fig. 2D). PDK1 is a key enzyme that regulates the fate of pyruvate by phosphorylating and thereby inhibiting pyruvate dehydrogenase (PDH). The effect of loss of PFKFB4 on PDK1 was verified using CRISPR mediated *PFKFB4* knockout with doxycycline inducible flag-tagged Cas9, which resulted in decreased levels of PDK1 protein and reduced PDH phosphorylation (Fig. 2E). Expression analysis of *PDK1* in the TCGA patient cohort also revealed a positive correlation with *PFKFB4* (*r* = 0.68) (Fig. 2F). This seemed to be specific to this PFK2/FBP2 isoform as the other isoforms expressed in glioblastoma (*PFKFB2* and *PFKFB3*) did not show any correlation with *PDK1* expression (Fig. S4D).

**Fig. 3:**
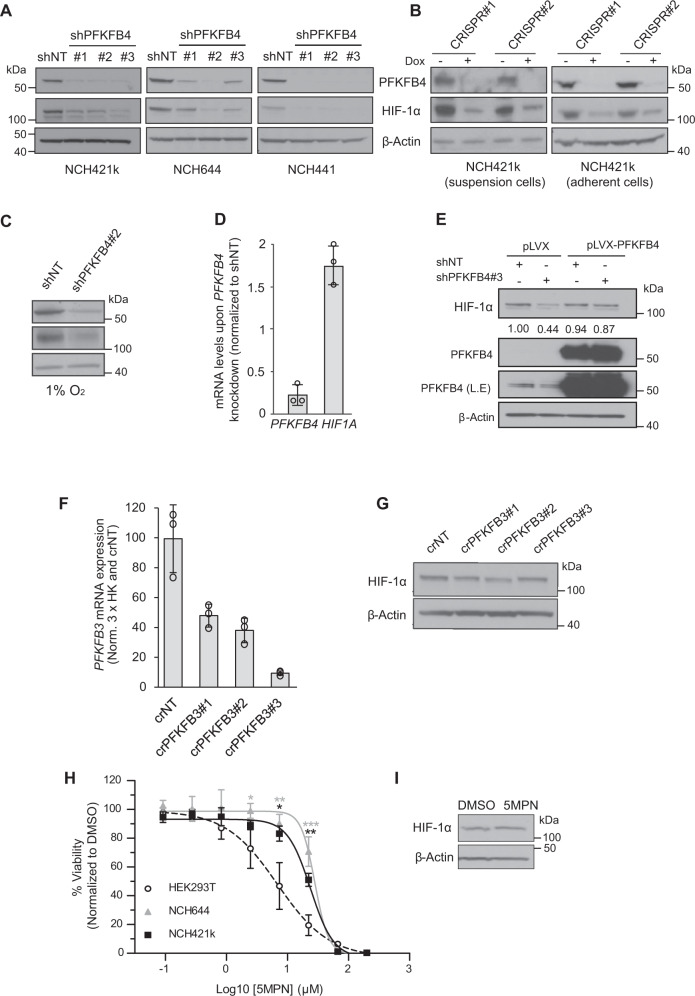
Characterization of HIF-1α regulation by PFKFB4. **A** Protein levels of PFKFB4 and HIF-1α upon silencing (3 days) of *PFKFB4* using three different shRNAs in NCH421k, NCH644 and NCH441 GSCs. β-actin was used as a loading control. **B** Protein levels of PFKFB4 and HIF-1α upon knockout of PFKFB4 using two different sgRNAs (7 days incubation with dox) in NCH421k GSCs grown in suspension and in an adherent monolayer. Knockout was induced by incubation with doxycycline for 7 days. β-actin is displayed as a loading control. **C** PFKFB4 and HIF-1α protein levels upon silencing of PFKFB4 in NCH421k GSCs cultivated in hypoxia (1% O_2_). β-actin is displayed as a loading control. **D** mRNA levels of *PFKFB4* and *HIF1A* upon silencing (3 days) of *PFKFB4* in NCH421k GSCs, normalized to housekeeper genes *HPRT*, *ARF1* and *DCTN2* and to shNT (*n* = 3; data are represented as mean ± SD). **E** Protein levels of PFKFB4 and HIF-1α in NCH421k GSCs which were transduced with lentiviral particles containing shNT or shPFKFB4#3, and empty pLVX-puro vector or pLVX-puro overexpressing PFKFB4. β-actin was used as a loading control. Numbers denote the quantified band intensity, normalized to β-actin and the first shNT band. L.E. = Longer exposure. **F** mRNA levels of *PFKFB3* upon knockout using 3 different guide RNAs (7 days after transduction and selection) in NCH421k GSCs, normalized to housekeeper genes *HPRT*, *ARF1* and *DCTN2* and to shNT (*n* = 3 technical replicates; data are represented as mean ± SD). **G** HIF-1α protein levels upon *PFKFB3* knockout. β-actin is displayed as a loading control. **H** IC_50_ curves of NCH421k and NCH644 GSCs or HEK293T cells after incubation with 5MPN at increasing concentrations for 48 hours (*n* = 3, mean ± SD, two sided *t*-test, *p value < 0.05, **p value< 0.01, ***p value< 0.001). **I** Protein levels of HIF-1α in NCH421k GSCs after incubation with 4 µM 5MPN for 72 hours. β-actin was used as a loading control.

Next, we evaluated the effect of PFKFB4 depletion on HIF-1α levels in a range of cell lines and conditions. A reduction in HIF-1α protein levels was observed in the three GSC lines grown as spheroids (Fig. 3A), or as an adherent monolayer (Fig. 3B, shown in NCH421k). *HIF1A* was silenced in NCH421k GSCs with two separate shRNAs to ensure the band observed between 100 and 140 kDa does indeed correspond to HIF-1α protein (Fig. S5A). Interestingly, HIF-2α protein was also depleted upon *PFKFB4* knockout (Fig. S5B). The growth of cancer cells in spheroids of over around 100 µm in diameter is known to cause the formation of an oxygen gradient, with the core of the spheroids becoming hypoxic [30]. Similarly, even in a monolayer, high cell confluency and seeding density can result in high oxygen consumption and the establishment of a hypoxic microenvironment [31]. Immunoblotting of hypoxia marker CA9 (Carbonic Anhydrase 9) in spheroids of different sizes suggested that hypoxia only arose in large spheroids (Fig. S5C–F). However, HIF-1α levels were similar in large (~250–450 µm) and small (<~50 µm) spheroids (Fig. S5E, F), indicating that the reduced HIF stability we observed upon PFKFB4 depletion was not due to differences in spheroid size. Furthermore, HIF-1α levels were also similar between dense and sparsely growing adherent GSCs (Fig. S5G, H). As HIFs are normally only stabilized when oxygen levels are low, it was interesting to also observe its stabilization in GSCs grown in small spheroids and in a sparse monolayer under normoxic conditions. Conversely, HIF-1α was hardly detected in HEK293T cells cultivated under normoxia, even when cells were cultivated in a very dense monolayer (Fig S5G, H). This suggests that HIF signaling is constituitively active in GSCs. Notably, a HIF-1α levels were also reduced upon PFKFB4 silencing in spheroids cultivated in hypoxia (1% oxygen) (Fig. 3C), suggesting that HIF-1α protein levels are dependent on PFKFB4 expression irrespective of oxygen availability.

Interestingly, mRNA levels of *HIF1A* did not decrease in GSCs when *PFKFB4* was silenced, and even seemed to become slightly higher (Fig. 3D). This indicates that the depletion of HIF-1α protein induced by *PFKFB4* silencing is not due to reduced transcription of *HIF1A* mRNA. Overexpression of PFKFB4 upon silencing of endogenous *PFKFB4* via an shRNA targeted to the 3’ untranslated region (UTR) (shPFKFB4_3) rescued HIF-1α protein levels, eliminating the possibility of off-target effects of the shRNAs on HIF-1α levels (Fig. 3E). Additionally, overexpression of PFKFB4 in HEK293T cells, in which endogenous PFKFB4 levels are very low, led to the stabilization of HIF-1α protein to a similar degree to the stabilization induced by hypoxia (Fig. S5I). Rate of cell proliferation was not affected (Fig. S5J). Notably, silencing of *PFKFB4* in other cancer cell lines cultivated under hypoxic conditions, such as DU145 (prostate) and MDA-MB-321 (breast), also caused a reduction of HIF-1α protein levels (Fig. S5K), suggesting that PFKFB4 could be important for the regulation of HIF-1α in additional cancer contexts to glioblastoma. PFKFB3 depletion did not affect HIF-1α expression in NCH421k GSCs (Fig. 3F, G).

We next sought to determine whether a currently available kinase inhibitor of PFKFB4, 5MPN [32], specifically targets GSCs and reduces HIF-1α levels. Suprisingly, GSCs were not sensitive to the compound compared with HEK293T cells, which do not express PFKFB4 under normoxia (Fig. 3H). Further, 5MPN did not lead to a reduction of HIF-1α protein levels in GSCs (Fig. 3I). This suggests that the effect of *PFKFB4* silencing on cell GSC viability and HIF-1α levels is independent of the kinase activity of PFKFB4.

### PFKFB4 interacts with FBXO28, an E3 ubiquitin ligase

**Fig. 4:**
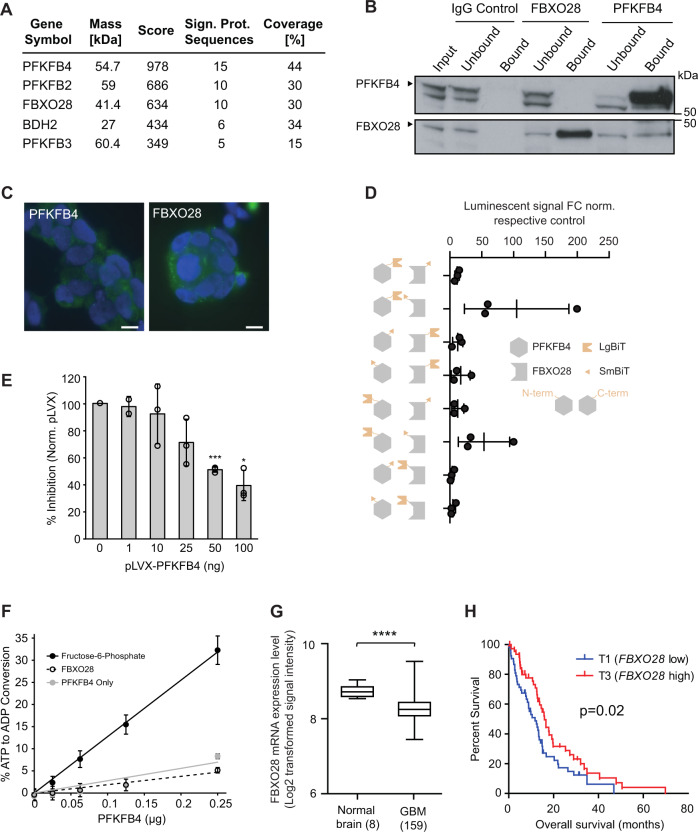
FBXO28 is a novel interaction partner of PFKFB4. **A** Interaction partners of PFKFB4 in NCH421k GSCs as determined by mass spectrometry of immunoprecipitated PFKFB4 from NCH421k lysate. Proteins were only considered as identified if more than one unique peptide had an individual ion score exceeding the MASCOT identity threshold and if three or more unique peptide sequences were identified in all replicates and not in the controls. The experiment was performed in biological triplicate, with data from one biological replicate displayed here; further replicates shown in Table S2. **B** Immunoprecipitation of FBXO28 or PFKFB4 from NCH421k lysate, with IgG as a control. PFKFB4 and FBXO28 are shown on the immunoblot. **C** Immunofluorescent staining of PFKFB4 and FBXO28 in NCH421k GSCs. Scale bar = 10 µm. **D** Split NanoLuc^®^ Luciferase assay to determine the best combination of split fragments with the large (LgBiT) or small (SmBiT) BiT cloned onto the C- or N-terminal of either PFKFB4 or FBXO28. Data are normalized to the negative control, which is a combination of the halo tag labelled with the small BiT with either PFKFB4 or FBXO28 tagged with the large BiT. A luminescent signal that is at least 10 times the negative control was considered as positive. Data are represented as mean of biological triplicates ± SD. **E** Verification of the specificity of the interaction of SmBiT-tagged FBXO28 (N-term) to LgBiT-tagged PFKFB4 (C-term) using the Split NanoLuc^®^ Luciferase system. Increasing amounts of untagged PFKFB4-overexpressing pLVX plasmid were added to displace the tagged protein, with empty pLVX plasmid as a negative control. Data are depicted as percent inhibition compared with the pLVX only control. (*n* = 3, mean ± SD, two sided *t*-test, *p value < 0.05, ***p value< 0.001). **F** ADP-Glo™ kinase assay measuring the percentage ATP to ADP conversion when increasing amounts of recombinant PFKFB4 was incubated with fructose-6-phophate, recombinant FBXO28 or no substrate. **G** *FBXO28* mRNA in normal brain (*n* = 8) compared with GBM patients (*n* = 159). Data are represented as mean ± the minimum and maximum. The whiskers are drawn down to the 25th percentile and up to the 75th percentiles. **H** Survival of GBM patients with high (top tertile, *n* = 53; red) and low (bottom tertile, *n* = 53; blue) *FBXO28* expression.

We then performed mass spectrometric analysis of PFKFB4 immunoprecipitated from NCH421k GSC lysate to find binding partners of PFKFB4 that could be involved in the stabilization of HIF-1α. Identified proteins are listed in Fig. 4a and Table S2. F-box Only Protein 28 (FBXO28) was of particular interest as it is an E3 ubiquitin ligase known to be part of the SCF complex formed by S-phase associated protein 1 (SKP1), cullin 1 (CUL1) and variable F-box proteins, involved in proteasomal degradation. We confirmed the binding of FBXO28 to SKP1 and CUL1 in a co-immunoprecipitation (co-IP) experiment in HEK293T cells (Fig. S6A). The binding of FBXO28 to PFKFB4 was verified by co-IP in GSCs and Yeast-Two-Hybrid assays (Figs. 4B and S6B). In addition, the cytoplasmic localization of both proteins in GSCs was confirmed by immunofluorescence (Fig. 4C). A split NanoLuciferase Binary Technology (BiT) reporter assay was developed in HEK293T cells to detect the interaction between PFKFB4 and FBXO28 tagged with a small and a large BiT fragment. 8 different combinations of PFKFB4 and FBXO28 with the large (LgBiT) and small (SmBiT) BiT tags on the N- and C- termini were tested to find the optimal binding conditions. The luminescence signal arising from the interacting tags brought into proximity with each other due to the binding of PFKFB4 and FBXO28 is shown in Fig. 4D. The specificity of the assay was assessed by overexpressing BiT-tagged FBXO28 and PFKFB4 in HEK293T cells together with increasing concentrations of untagged PFKFB4, acting as a competitor for tagged PFKFB4 (Fig. 4E). In order to address whether PFKFB4 regulates the activity of FBXO28 by phosphorylation, we performed an in vitro kinase assay with recombinant PFKFB4 and FBXO28, using the ADP-Glo™ kinase assay to determine the amount of ATP converted to ADP during the reaction (Fig. 4F). The level of ATP to ADP conversion was similarly low with or without recombinant FBXO28, whereas up to 36% ATP to ADP conversion was observed when PFKFB4 was incubated with its known metabolic substrate, fructose-6-phosphate. This indicates that PFKFB4 does not phosphorylate FBXO28, and could suggest that the regulation of FBXO28 by PFKFB4 occurs via a physical blocking of the interaction of FBXO28 with its substrate. FBXO28 has been previously shown to regulate MYC activity in cancer cells, and was found in the same study to be upregulated on the mRNA level in various tumor entities [33]. In order to determine whether PFKFB4 may affect MYC activity via its interaction with FBXO28, we performed GSEA on our *PFKFB4*-silenced ranked gene list (see Fig. 2) using published gene signatures of MYC-activated and repressed targets [34]. There was no significant enrichment for up- or downregulation of MYC target genes upon *PFKFB4* silencing (Fig. S7), leading us to conclude that PFKFB4 does not affect the regulation of MYC in GSCs. Interestingly, gene expression profiling data from glioblastoma patients shows that FBXO28 is actually downregulated in glioblastoma compared with normal brain, and low FBXO28 expression correlates with poor survival [35] (Fig. 4G, H).

**Fig. 5:**
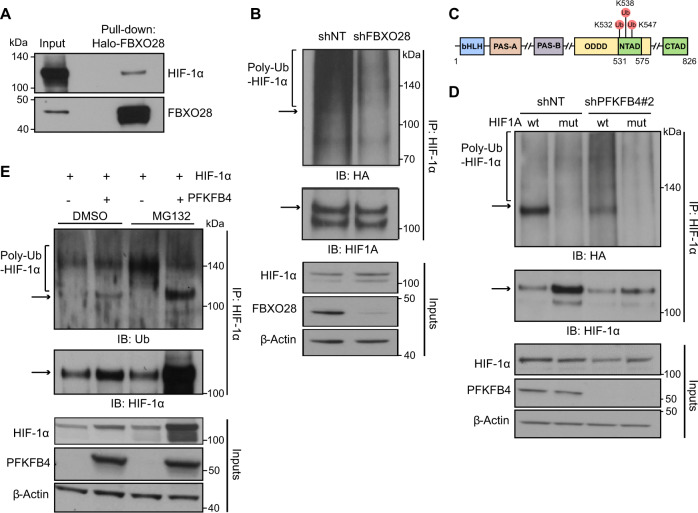
PFKFB4 hinders the ubiquitylation of HIF-1α by binding to the E3 ubiquitin ligase FBXO28. **A** FBXO28-Halo and HIF-1α were overexpressed in HEK293T cells, and an FBXO28 pulldown was performed using Halo resin. FBXO28 and associated proteins were eluted by TEV-cleavage. Immunoblot was performed with FBXO28 and HIF-1α antibodies. **B** Upper panel: HIF-1α IP from lysate of NCH421k cells stably expressing HA-ubiquitin, in the presence of the proteasome inhibitor MG132 (500 nM, 6 hours) and with and without *FBXO28* silencing (3 days). The immunoblot (IB) shows HA-ubiquitin and HIF-1α levels. The bracket indicate poly-ubiquitinated HIF-1α and the arrow shows the expected migration of unmodified HIF-1α (also applies to 5D and 5E). Lower panel: HIF-1α and FBXO28 protein levels in the input lysates used in the IP above. β-actin is displayed as a loading control in all panels. **C** Scheme of the HIF-1α protein domains including the ubiquitinylated lysines which were mutated to alanine (K532, K538, K547) (Adapted from ref. [54]). **D** Upper panel: HIF-1α IP from lysate of NCH421k cells stably expressing HA-ubiquitin and silenced (3 days) for endogenous *HIF1A*, in the presence of MG132 (500 nM, 6 hours), with and without *PFKFB4* silencing and overexpression of wild-type or lysine-mutated HIF-1α. The IB shows HA-ubiquitin and HIF-1α levels. Lower panel: PFKFB4 protein levels in the input lysates used in the IP above. **E** Upper panel: HIF-1α immunoprecipitated (IP) from lysate of HEK293T cells in the absence and presence of MG132 (500 nM, 6 hours) and PFKFB4 overexpression (3 days). HIF-1α was overexpressed in all conditions. The IB shows ubiquitin (Ub) and HIF-1α levels. Lower panel: PFKFB4 protein levels in the input lysates used for the IP above.

The identification of the E3 ubiquitin ligase component FBXO28 as an interaction partner of PFKFB4 led us to hypothesize that FBXO28 may regulate the ubiquitylation and subsequent proteasomal degradation of HIF-1α under regulation by PFKFB4. In line with this, in a pull-down assay in HEK293T cells overexpressing Halo-tagged FBXO28 and HIF-1α, FBXO28 was found to directly bind to HIF-1α (Fig. 5A) and silencing of *FBXO28* in NCH421k cells expressing HA-tagged ubiquitin (NCH421k-HA-Ub) caused a reduction in ubiquitylation of immunoprecipitated HIF-1α (Fig. 5B), providing evidence that HIF-1α is indeed a substrate for FBXO28 in GSCs. In order to investigate the effects of PFKFB4 depletion on HIF-1α ubiquitylation, *PFKFB4* was silenced in NCH421k-HA-Ub GSCs expressing wildtype HIF-1α or a triple lysine mutated HIF-1α variant (lysine residues whose ubiquitylation is known to be important for proteasomal degradation, K532R, K538R and K547R [36], were mutated to arginine (Fig. 5C)). Endogenous *HIF1A* was also silenced using an shRNA directed to the 3’ UTR (Fig. SX, shHIF1A#1). Although unmodified HIF-1α levels were reduced, the silencing of *PFKFB4* in cells overexpressing wildtype HIF-1α increased levels of poly-ubiquitylated HIF-1α. This was not the case in cells expressing mutant HIF-1α (Fig. 5D). Conversely, investigation of PFBFB4 expression in an endogenous PFKFB4-negative system (HEK293T cells) in which HIF-1α was over-expressed revealed an accumulation of poly-ubiquitylated HIF-1α when proteasomal degradation was inhibited, which appeared to be alleviated upon expression of PFKFB4, despite an apparent increase in overall HIF-1α protein levels (Fig. 5E). Taken together, this suggests that PFKFB4 can repress the activity of an E3 ubiquitin ligase which acts on HIF-1α, and further shows that the regulation of HIF-1α by PFKFB4 is indeed on the level of ubiquitylation and proteasomal degradation.

### The regulation of HIF-1α by PFKFB4 and FBXO28 contributes to GSC survival

**Fig. 6:**
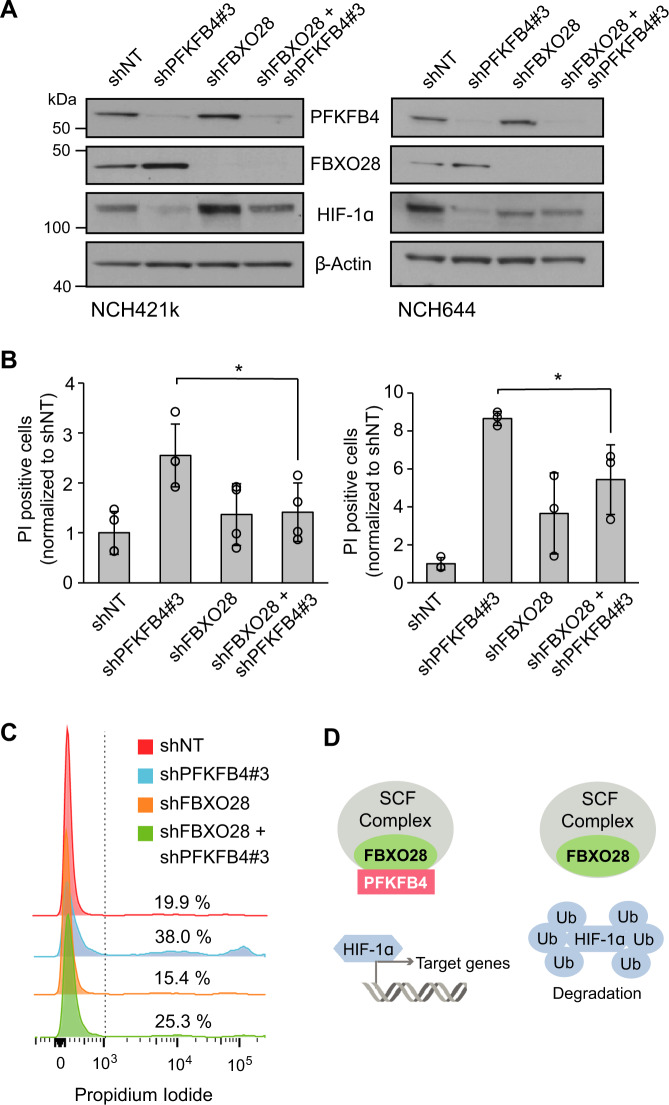
HIF-1α protein level is dependent on the interaction of PFKFB4 with FBXO28. **A** Protein levels of PFKFB4, FBXO28 and HIF-1α in NCH421k and NCH644 GSCs with *PFKFB4* and *FBXO28* silencing (3 days) separately or together. β-actin is displayed as a loading control. **B** FACS analysis of propidium iodide stained NCH421k (left) and NCH644 (right) GSCs with *PFKFB4* and *FBXO28* silencing (4 days) separately or simultaneously. Data are normalized to cells transduced with shNT (*n* = 3) and are represented as mean ± SD, two-sided *t*-test, *p value < 0.05. **C** Representative histograms of the FACS data highlighting the rescue of the phenotype upon simultaneous silencing of PFKFB4 and FBXO28. **D** Scheme showing the proposed mechanism of PFKFB4 and FBXO28 acting on HIF-1α stability and therefore cell viability (Ub = ubiquitin).

Next, we verified the importance of the interplay between FBXO28 and PFKFB4 for HIF-1α protein expression and its impact on GSC survival by determining whether FBXO28 is necessary for the cell death effect of *PFKFB4* silencing on GSCs. Concordant with the hypothesis that PFKFB4 suppresses the ability of the SCF complex to target HIF-1α for proteasomal degradation, silencing *PFKFB4* in NCH421k and NCH644 GSCs decreased the protein levels of HIF-1α, while silencing *FBXO28* led to similar or in NCH421k even increased HIF-1α levels. Silencing of both *FBXO28* and *PFKFB4* in GSCs rescued HIF-1α protein levels, with the amount detected comparable to the GSCs transduced with NT shRNA (Fig. 6A). Critically, FACS analysis of cell death using propidium iodide (PI) staining revealed that the cell death induced by *PFKFB4* silencing was also significantly decreased when *FBXO28* was silenced simultaneously (Fig. 6B, C), indicating that FBXO28 is indeed required for the degradation of HIF-1 α and subsequent cell death in GSCs upon silencing of *PFKFB4*. These results emphasize the novel role of PFKFB4 to protect HIF-1α from the SCF complex, enabling the expression of HIF-1α target genes and survival in glioblastoma stem-like cells (Fig. 6D).

## Discussion

The current standard treatment for glioblastoma using chemo- and radiotherapy targeting the proliferative bulk does not cure glioblastoma patients. One reason for this is the presence of glioblastoma stem-like cells, which do not respond to therapy in the same way as the rest of the tumor. Here we have shown that *PFKFB4* silencing greatly attenuates tumor growth in an orthotopic xenograft mouse model using patient derived GSCs, dramatically increasing survival. We then sought to further understand the cellular function of PFKFB4 in GSCs in a bid to harness the protein as a therapeutic target. Gene expression profiling of *PFKFB4*-silenced GSCs revealed the involvement of hypoxia inducible factors (HIFs), and Western blot analyses showed that HIF protein levels are influenced by PFKFB4. We identified E3 ubiquitin ligase FBXO28 as a novel binding partner of PFKFB4, and have shown that the regulation of HIF-1α protein levels by PFKFB4 is mediated by the ubiquitin ligase activity of FBXO28.

Interestingly, we found that HIF-1α protein is upregulated in GSCs even under normoxic conditions. HIF-1α has been shown to be upregulated in various solid malignancies, with upregulation often corresponding to tumor malignancy [37–39]. Increased oncogenic signaling via the PI3K/Akt/mTOR and MAPK/ERK pathways, along with increased reactive oxygen species (ROS) production have been shown to contribute to the metabolic reprogramming of glioma cells, leading to constitutive HIF signaling (reviewed in ref. [40]). Downregulation of proteins mediating HIF-1α degradation, such as FBXO28, could provide these tumor cells with a further mechanism for this upregulation. Our finding that FBXO28 is downregulated in glioblastoma patients is in line with this. Indeed, another E3 ubiquitin ligase F-box/WD repeat-containing protein 7 (FBW7), one of the most frequently mutated genes in human cancer and well known tumor suppressor [41], has been shown to target HIF-1α for proteasomal degradation in hypoxic conditions [42] and is also downregulated in glioblastoma patients compared with normal brain.

The mechanism we propose of PFKFB4 controlling the stability of HIF-1α is somewhat removed from the traditional metabolic function [16, 18, 43] of the enzyme. However, as HIF-1α regulates the transcription of many genes which allow the cell to adapt to hypoxic stress, including genes involved in aerobic glycolysis [44], the effect of *PFKFB4* disruption on glucose metabolism might be exacerbated by the down-regulation of HIF-1α target genes that are directly involved in metabolism. As HIF-1α has also been shown to regulate the expression of *PFKFB4* [45] a positive feedback loop for the expression of both PFKFB4 and HIF-1α is likely at play, supporting the high levels of both of these proteins observed in GSCs. We propose that GSCs are dependent on this feedback loop and that the dramatic cell death in GSCs induced by *PFKFB4* silencing is due to the effects of HIF depletion. This is supported by previous studies demonstrating that HIF-1α and HIF-2α are critical for GSC maintenance and stem cell phenotype [46, 47]. Although we focused on HIF-1α in this study, we could also show that PFKFB4 depletion led to reduced HIF-2α levels. Disrupting only one HIF isoform can lead to compensation by other isoforms and therefore a loss of efficacy. This was recently reported in a study testing the specific HIF-2α inhibitor PT2385 in glioblastoma, whereby the treatment did not alter cellular phenotype [48]. Although there has been increased interest in intervening with HIF-1α in recent years, the complexity of the pathway has made the rational design of inhibitors challenging [49] and the pan-HIF inhibitors that have been developed tend to have dose-limiting side effects that may prevent their use as chemotherapeutic agents [25]. However, a phase II clinical trial of the specific HIF-2α inhibitor PT2385, mentioned above, has indicated that this drug was well tolerated by patients (NCT03216499) [50].

After identifying E3 ubiquitin ligase, FBXO28, as an interaction partner of PFKFB4, we found that FBXO28 binds to HIF-1α, and that PFKFB4 regulates HIF-1α ubiquitination. Importantly, we could also show that FBXO28 is required for the effect of PFKFB4 silencing on HIF-1α levels and cell death in GSCs. This suggests that PFKFB4 regulates HIF-1α stability via the repression of the ubiquitin ligase activity of FBXO28. It would be interesting to further investigate whether HIF-2α is also a substrate for FBXO28, or whether there are other E3 ubiquitin ligases at play which are regulated by PFKFB4. However, GSCs were not sensitive to kinase inhibition of PFKFB4 with 5MPN compared with HEK293T cells, nor did 5MPN treatment lead to a reduction of HIF-1α protein levels. As HEK293T cells do not express PFKFB4 in normoxia, it seems that the cell death caused by the compound at high µM concentrations was due to unspecific or off-target effects. This suggests that the role of PFKFB4 in regulating HIF-1α levels and thereby GSC survival is independent of its kinase activity, and is supported by our finding that PFKFB4 does not phosphorylate FBXO28 in vitro. As PFKFB4 has both kinase and phosphatase functions, it remains to be seen whether dephosphorylation is at play. Although we provide evidence for the importance of the interaction of PFKFB4 and FBXO28 in the regulation of HIF-1α, the interaction has yet to be mapped. This will be an important step which will aid the rational design of compounds which disrupt this protein-protein interaction. Given the cancer specificity of PFKFB4, such compounds could open up a whole new avenue for inhibiting HIF-1α in a cancer specific manner.

## Material and methods

### Primary patient derived GBM models

The GSC lines NCH421k, NCH441 and NCH644 were derived from primary GBM patients who underwent surgical resection according to the research proposals approved by the Institutional Review Board at the Medical Faculty of Heidelberg. Tissues were enzymatically dissociated and cells were cultivated as floating neurospheres in serum-free DMEM/F-12 medium (Merck Millipore, F4815), supplemented with 20% BIT-admixture (Pelo Biotech, PB-SH-033-0000) and basal fibroblast growth factor (Biomol, 50361.50) and epidermal growth factor (Life Technologies, PHG0311) at 20 ng/ml each. Genotypic and phenotypic studies have been carried out in previous investigations [51, 52]. Patient information is listed in Table S3. Passaging and seeding was carried out by dissociation with accutase (Sigma-Aldrich, A6964-100ML). All experiments were performed using GSCs grown as neurospheres unless otherwise stated. Typically, neurospheres reach around 200–300 µm in diameter after culturing for 4 days. Adherent culturing of GSCs followed in Poly-L-lysine (30 µg/ml) (Sigma-Aldrich, P9155-5MG) and laminin (7.5 µg/ml) (Sigma-Aldrich, L2020-1MG) coated flasks. All cells were cultured in a standard tissue culture incubator maintained at 37 °C with 95% humidity and 5% CO_2_. All cell culture and experimental work was performed in normoxic conditions (21% O_2_) unless otherwise stated.

### Established cell lines

HEK293T, U373, SW480 and MDA-MB-231 cells were cultured adherently in DMEM (Gibco, 11965092) supplemented with 10% FCS and 1% (v/v) penicillin/streptomycin under standard conditions. Cells were passaged using trypsin-EDTA (Sigma-Aldrich, T3924-100ML). Cell lines were authenticated using the in-house service of the DKFZ and tested bi-annually for mycoplasma at GATC.

### Lentiviral production and transduction

HEK293T cells were seeded into 10 cm dishes and co-transfected with the plasmid of interest and the packaging plasmids (pMD2.G and psPAX2 (Addgene plasmids # 12259 and 12260)) using Trans-IT transfection reagent (Mirus, 731-0028). Virus containing medium was collected and filtered 72 hours after transfection and concentrated by ultracentrifugation for 1.5 hours at 25,000 rpm. The titer was determined by FACS using the plko.1-TurboGFP control plasmid (Sigma-Aldrich, SHC003). For transduction, cells were seeded at a density of 50,000 cells per ml and transduced with a multiplicity of infection (MOI) of 5. plko.1-TurboGFP, containing lentivirus was used to estimate transduction efficiency via FACS. Experiments were discarded if <70% of cells were positive for GFP. For generation of stable cell lines, transduced cells were selected with 2 µg/ml puromycin (Millipore, 540411-25MG) or 400 µg/ml G418 (Calbiochem, 345810), depending on the resistance cassette of the construct.

### Gene set enrichment analysis

GSEA analysis was performed at www.broadinstitute.org/gsea. Genes were pre-ranked based on the mean fold change upon PFKFB4 silencing and analysed for the enrichment of the CGP: chemical and genetic perturbations signatures (3343 gene sets), as listed in the Molecular Signatures Database (http://www.broadinstitute.org/gsea/msigdb). Quantification of enrichment of the signature genes near the top of the ordered list of genes followed using a running-sum statistic, resulting in an enrichment score. The FDR q-value was used to set a significant threshold.

### Propidium iodide staining

GSCs were dissociated with 50 µl accutase and, after washing with PBS, resuspended in 200 µl of a 5% FBS, 0.5% propidium iodide (PI) in PBS solution. PI positive cells were measured using a FACSCanto II and quantified with FACSDiva software (version 6.3.3).

### IC_50_ determination

GSCs or HEK293T cells were seeded in 96-well plates at a density of 10,000 or 3000 cells per well respectively. 5MPN (Sigma Aldrich) was diluted in DMSO and applied at various concentrations. Viability was measured after 48 hours using the CellTiter-Glo assay (Promega), according to the manufacturer’s protocol and using a Mithras LB 940 plate reader (Berthold Technologies). IC_50_ values were determined by applying a four parameter logistic curve using Graphpad Prism 8.

### In vitro kinase assay

0, 0.1, 0.25, 0.5 and 1 µg recombinant PFKFB4 (71199, BPS Bioscience) was incubated for 30 min at 30 °C with 0.25 µg FBXO28 (TP760267, OriGene), 1 mM fructose-6-phopshate (Sigma) or no substrate (control) in the presence of 20 µM ultrapure ATP in 1 x kinase buffer (Cell Signaling, #9802). 5 µl of each reaction was analysed by the ADP-Glo™ Kinase Assay (Promega, V6930) in a 384-well plate according to the manufacturer’s protocol. The percentage of ATP to ADP conversion was calculated using a standard curve of known ATP + ADP concentrations. Luminescence signal was measured using a Mithras LB940 plate reader (Berthold Technologies).

### Statistics

Statistical testing was carried out using the functions in Excel 2016, or in GraphPad Prism 5. To allow for testing, unless otherwise stated, experiments were carried out in biological triplicate and data is represented as the mean ± standard deviation. Unpaired two sided student’s *t*-testing was used to compare means. *p*-values were represented as follows: *p < 0.05, **p < 0.01, ***p < 0.001. “n.s.” denoted differences in means that were not significant. In general, variance was roughly equal between groups. If variance between groups was different, such as when one group was normalized, the unequal variance (Welch’s) unpaired two sided student’s *t*-test was used. The differences in the distribution of survival data in Kaplan Meier analyses was evaluated using the log rank (Cox-Mantel) test. The TCGA GBM-LGG RNAseq V2 RSEM-normalized expression matrix was downloaded from the Broad TCGA GDAC (https://confluence.broadinstitute.org/display/GDAC/Home), filename: gdac.broadinstitute.org_GBMLGG.Merge_rnaseqv2__illuminahiseq_rnaseqv2__unc_edu__Level_3__RSEM_genes_normalised__data.Level_3.2016012800.0.0, and filtered for the *n* = 152 GBMs. The following analysis was performed in R v3.5.1. Correlation plots were created with ggplot2 (v3.1.0). The activation of the HIF-related signature was calculated using the ssGSEA implementation in GSVA (v1.30.0) for each GBM sample in the filtered RNAseq expression matrix. Trend lines were inferred using a linear model.

Details about the following methods are included as supplementary information: Orthotopic brain tumor xenograft model, tumor microarray, shRNA constructs, CRISPR constructs, overexpression plasmids, development of monoclonal antibodies against PFKFB4, immunohistochemistry, immunofluorescence of cells, microarray analysis, RNA extraction and, quantitative real-time PCR analysis, immunofluorescence of paraffin sections, immunoprecipitation, protein elution and tryptic digestion for mass spectrometry, mass spectrometry and database searches, split NanoLuc® luciferase system, yeast two hybrid, Western blot analysis.

## Supplementary information

Supplementary Methods

Supplementary Figures

Supplementary Tables

## Data Availability

The gene expression profiling data have been submitted to the Gene Expression Omnibus under accession GSE108742. The mass spectrometry proteomics data have been deposited at the ProteomeXchange Consortium via the PRIDE [53] partner repository with the dataset identifier PXD009807 and 10.6019/PXD009807. Other data that support the findings of this study are available from the corresponding author upon request.
