# Supplementary material for: PFKFB4 interacts with FBXO28 to promote HIF-1α signaling in glioblastoma
Source: Oncogenesis. 2022 Sep 17;11(1):57. doi: 10.1038/s41389-022-00433-3 (PMC9482633; doi:10.1038/s41389-022-00433-3)
Supplement: Supplementary file 1 — Supplementary Methods [file 41389_2022_433_MOESM1_ESM.pdf]

PFKFB4 interacts with FBXO28 to promote HIF-1α signaling in glioblastoma

Emma Phillips[1], Jörg Balss[1], Frederic Bethke[1], Stefan Pusch[2,3], Stefan Christen[4,5], Thomas Hielscher[6], Martina Schnölzer[7], Michael N. C. Fletcher[8], Antje Habel[2], Claudia Tessmer[9], Lisa-Marie Brenner[1], Mona Göttmann[1], David Capper[2,3,10], Christel Herold-Mende[11], Andreas von Deimling[2,3], Sarah-Maria Fendt[4,5], and Violaine Goidts[1]

Supplementary Methods

Orthotopic Brain Tumor Xenograft Model

NCH421k GSCs were transduced with lentiviral particles carrying the pLenti PGK V5-LUC Neo plasmid, which was a gift from Eric Campeau (Addgene plasmid # 21471) (1) and transduced cells were selected using 400 µg/ml G418. shPFKFB4_2 and shNT were cloned into the pLKO-Tet-ON vector and lentiviral particles produced as described below. NCH421k cells were transduced and selected under 2 µg/ml puromycin. Six week old female NOD-SCIDγ mice were obtained from the animal facility at the DKFZ. All animal experiments were performed according to animal welfare regulations and were approved by the responsible authorities (Regierungspräsidium Karlsruhe, approval number G13-13). Animal group sizes were determined based on tumor growth kinetics, which was determined in a pilot study. NCH421k luciferase cells containing genes for the inducible expression of shPFKFB4_2 or shNT were treated with accutase and 100 000 cells were injected into the striatum of the mice in a volume of 4 µl. 8 mice were injected with shNT cells and 14 mice were injected with shPFKFB4_2 cells. Tumor growth was monitored using an IVIS Lumina Pre-clinical in vivo Imaging System and Living Image Software (version 2.50.1) (Perkin Elmer) twice a week for 6 weeks or until the development of neuropathological symptoms. 2 mg/l doxycycline was administered via drinking water after tumors had reached radiances of around 200 000 flux p/s. A control group of mice with shPFKFB4_2 tumors were not treated with doxycycline. The animals were

assigned to the groups alternately based on when the tumor reached the threshold – i.e. mouse 1 -> dox, mouse 2 -> no dox, mouse 3 -> dox, mouse 4 -> no dox, etc. Within the dox treatment group, the experimental investigator was blinded as to whether each animal had a shNT or shPFKFB4 expressing tumor to ensure unbiased decision making in end point determination.

Tumor Microarray

The material used for the tumor microarray was provided by the tissue bank of the NCT Heidelberg in accordance with the tissue bank regulations and permission of the ethics commission of the University of Heidelberg. Two independent investigators performed scoring of PFKFB4 expression, and were blinded to the tumor grade.

shRNA Constructs

pLKO.1 containing shRNA plasmids were purchased from Sigma Aldrich. shRNA sequences are listed in Table S4.

CRISPR Constructs

For PFKFB4 knockout, sgRNAs were designed using the CHOP CHOP tool (http://chopchop.cbu.uib.no) and cloned into a modified version of the lentiguide-puro v2 plasmid, in which the puromycin resistance cassette was switched for neomycin resistance. The lentiguide-puro v2 plasmid was a gift from Feng Zhang (Addgene plasmid # 52961) (2). sgRNA sequences are listed in Table S5. NCH421k GSCs were transduced with lentiviral particles containing the pCW-Cas9 doxycycline inducible FLAG-Cas9 plasmid, which was a gift from Eric Lander & David Sabatini (Addgene plasmid #50661) (3), and positively transduced cells selected for using 2 µg/ml puromycin. Cells were then transduced with the sgRNA containing lentiguide-Neo lentiviral particles and positively transduced cells selected for using 400 µg/ml G418. To induce the expression of the Cas9 plasmid, cells were incubated with 1 µg/ml doxycycline for 7 days, and doxycycline was replenished in the medium every two days.

For PFKFB3 knockout, sgRNAs were designed as above and cloned into the lentiCRISPRv2 blast plasmid, which was a gift from Brett Stringer (Addgene plasmid #98293). NCH421k GSCs were transduced with the sgRNA containing lentiCRISPRv2 lentiviral particles and positively transduced cells selected for using 10 µg/ml blasticidin. After 7 days, cells were harvested and analyzed for PFKFB3 mRNA levels and HIF-1α protein levels.

Overexpression Plasmids

PFKFB4, FBXO28, CUL1 and SKP1 were amplified from cDNA and cloned into the pLVX lentiviral vector. For the split NanoLuc® luciferase assay, PFKFB4 and FBXO28 were cloned into the NanoBiT® vectors and the protein sequences with the tags were subsequently cloned into the pLVX lentiviral vector. Primers are listed in Table S6. For overexpression of HIF-1α, the HA-HIF1alpha-pcDNA3 plasmid was used, which was a gift from William Kaelin (Addgene plasmid #18949) (4). In order to allow its overexpression in GSCs, HIF1A was cloned into the pLVX vector. Mutations of HIF1A _K532R, K538R, K547R (HIF1A_mut) were performed using the Q5® Site-Directed Mutagenesis Kit (NEB #E0554S), following the manufacturer’s protocol. Primers are listed in Table S7. For overexpression of HA-ubiquitin, the pLenti puro HA-Ubiquitin plasmid was used, which was a gift from Melina Fan (Addgene plasmid #74218).

Development of monoclonal antibodies against PFKFB4

The PFKFB4 full length protein was expressed as GST-fusion protein in SF9 insect cells. After expression the protein was purified by affinity chromatography (Glutathione Sepharose) and the Tag was removed by cleavage with Tobacco Etch Virus protease, followed by size exclusion chromatography. Mice (BALB/c or C57BL/6N) were immunized with the tag-free native, recombinant, human full-length PFKFB4. Sero-positivity of peripheral blood samples of the different animals was verified by WB. The best responding animals were selected for hybridoma fusion according to the principles of Köhler and Milstein's hybridoma technology (5). 820 supernatants were screened by ELISA (with purified, full-length protein). 104 clones

positive for PFKFB4 were further verified via immunofluorescence and Western blot using the glioblastoma adherent cell lines U87 and the GSC NCH421k lines. Validated mother clones were subcloned by limited dilution to obtain monoclonal cell clones.

Immunohistochemistry

Mice were sacrificed by CO_2 asphyxiation. Brains were extracted and perfused with 10 % (w/v) formaldehyde. They were then sectioned coronally, dehydrated in a STP 120 spin tissue processor and embedded in paraffin. Sections of 4 µm thickness were cut and mounted onto glass slides, deparaffinized in xylene and rehydrated in a descending series of alcohols. Antigen retrieval was performed by incubation in a steamer for 30 minutes in CC1 buffer (pH 8.0). Blocking ensued in 3 % H_2O_2/TBS for 60 min at room temperature. Slides were treated with primary antibody overnight at 4°C and incubated with a HRP conjugated secondary antibody for 1 h at room temperature. Staining was visualized using the Dako REAL Detection System and freshly prepared diaminobenzidine as a chromogen. Slides were counterstained with hematoxylin, dehydrated and mounted. Images were made using a Zeiss Axioplan 2 microscope.

Immunofluorescence of cells

GSCs were grown adherently on coverslips. Cells were fixed for 15 min with 4 % FA and permeabilised for 15 min with 0.1 % Triton-X. After blocking for 1 hour with 0.2 % BSA, the primary antibody (PFKFB4 1:100; FBXO28 1:100) was applied in blocking solution overnight at 4°C. Secondary antibodies were applied at 1:500 for 2 hours at room temperature. Coverslips were mounted onto slides using hardset mounting medium with DAPI (Vector Laboratories).

Microarray Analysis

NCH421k, NCH441 and NCH644 cells were transduced with shNT and shPFKFB4_2. After 4 days, RNA was extracted from cells using the RNAeasy Mini Kit (Qiagen) according to the

manufacturer's protocol and examined for integrity using an Agilent 2100 Bioanalyzer. Gene expression profiling was performed using the 4 x 44K Whole Human Genome Oligo Microarray (Agilent Technologies). Microarray slides were scanned on an Agilent G2505C Microarray Scanner and data extracted using the Agilent Feature Extraction software version 9.1.

RNA Extraction and Quantitative Real-time PCR Analysis

1 µg RNA was pre-treated with DNase I (Life Technologies) and reverse transcribed by Superscript II (Life Technologies) according to the manufacturer's protocol. Complementary cDNA was measured using Absolute SYBR Green ROX Mix (ABgene) in technical triplicate using an ABI PRISM 7900HT thermal cycler (Applied Biosystems). cDNA levels were quantified based on the standard curves based on a serial dilution of cDNA transcribed from NCH421k RNA and normalization to the housekeeper genes *ARF1*, *DCTN2* and *HPRT*. Oligonucleotide sequences are detailed in Table S8.

Immunofluorescence of paraffin sections

For PFKFB4 immunofluorescence detection in paraffin embedded tissue, sections were deparaffinized in xylene and rehydrated in a descending series of alcohols. Antigen retrieval was performed by incubation in a steamer for 30 minutes in CC1 buffer (pH 8.0). Blocking ensued in 3 % H_2O_2/TBS for 60 min at room temperature. The TSA kit #2 (Thermofisher) was used. Anti-PFKFB4 was used at a dilution of 1:25 and incubated in 1 % blocking reagent at 4°C overnight. Secondary antibodies were incubated at a 1:100 dilution in 1 % blocking buffer for 1 hour at room temperature, and tertiary fluorescent antibodies were subsequently incubated at a 1:100 dilution in 0.0015 % H_2O_2 in amplification buffer for 30 min at room temperature. Nuclei were stained using DAPI containing Vectashield (Vector Labs). Images were made using a Zeiss Axioplan 2 microscope.

Immunoprecipitation

For finding PFKFB4 binding partners and verifying the interaction between PFKFB4 and FBXO28, NCH421k GSCs were grown to confluence (around 25 million cells in 25 ml) in a T150 flask. Cells were lysed for 30 min on ice with intermittent vortexing using co-immunoprecipitation (Co-IP) buffer (Thermo Scientific). Protein concentration was measured by BCA assay, and the lysate was precleared for 1 hour at 4°C with 20 µl magnetic Dynabeads (Life Technologies) slurry. 1 µg PFKFB4 or FBXO28 antibody, or 1 µg mouse IgG as a negative control, was bound to 20 µl beads by incubation in PBS-Tween (0.02%) for 20 min at room temperature. 500 – 750 µg lysate was applied to antibody or IgG bound beads, and incubated on a wheel at 4°C overnight. Beads were washed 3 times with co-IP buffer and protein was eluted by incubation for 10 min at 70°C with 2 x LDS loading dye and reducing agent (Life Technologies). 40 µg total extract was loaded onto the gel as the "input," along with all the eluted protein from the beads as the "IP."

For showing the interaction between FBXO28 and CUL1 and SKP1, HEK293T cells grown in T150 flasks were transfected with pLVX constructs for the overexpression of each protein. Transfection followed using 8 µg of plasmid with 24 µg PEI (Polysciences Inc). Cells were harvested 3 days after transfection. The co-IP protocol was followed as detailed above, incubating 1 µg of the relevant antibody or mouse or rabbit IgG as a negative control.

For investigating the effects of PFKFB4 on HIF1A ubiquitination, HEK293T cells were transfected with HA-HIF1alpha-pcDNA3 plasmid and pLVX_PFKFB4 plasmid depending on the experimental set-up. 2 days after transfection, cells were incubated with 500 nM MG132 (Sigma-Aldrich) for 6 hours. Cells were harvested and immunoprecipitation of HIF-1α followed as detailed above. For studies in NCH421k cells, cells were transduced with viral particles containing the pLenti puro HA-Ubiquitin plasmid and a stable cell line was generated. 5 million cells were seeded into T150 flasks and transduced with shNT, shFBXO28, shPFKFB4_2 and/or shHIF1A, as well with pLVX_HIF1A wild-type or mutant containing lentiviral particles. 3 days after transduction, cells were incubated with 500 nM MG132 (Sigma-Aldrich) for 6 hours. Cells were harvested and immunoprecipitation of HIF-1α followed as detailed above.

Protein Elution and Tryptic Digestion for Mass Spectrometry

Beads were washed with PBS and washing solutions were discarded. For protein elution beads were incubated in 20 µL LDS sample buffer (20 µL 4xLDS, 8 µL reducing agent, 52 µL H2O) at 70°C for 10 min. The supernatant was loaded on a 4-12 % BisTris Gel (Invitrogen) in MES buffer and a short (1cm) electrophoresis run was performed at 200 V, 300 mA, 50 W for 5 min 35 sec. The gel was stained with colloidal Coomassie for 3 hours and the stained parts of the gel lanes were excised. Proteins in the individual gel slices were reduced with DTT, alkylated with iodoacetamide and in-gel digested with trypsin (Promega) overnight. After collecting the supernatant gel pieces were sonicated for 5 min in 0.1% trifluoroacetic acid followed by extraction with acetonitrile. After three further extraction steps the combined supernatants were dried in a speed-vac concentrator at 37°C for 2 hours. Peptides were dissolved in 5 µl 0.1% TFA/2.5% hexafluoro-2-propanol by sonication for 5 min and subsequently analyzed by nanoLC-ESI-MS/MS.

Mass spectrometry and Database Searches

Peptide mixtures were separated using a nanoAcquity UPLC system. For trapping we used a C18 pre- column (180 µm × 20 mm) with a particle size of 5 µm (Waters GmbH, Eschborn, Germany). Liquid chromatography separation was performed on a BEH130 C18 main-column (100 µm × 100 mm) with a particle size of 1.7 µm (Waters GmbH, Eschborn, Germany). Peptide mixtures were loaded on the trap column at a flow rate of 5 µl/min and were eluted with a gradient at a flow rate of 400 nL/min. Chromatography was carried out using a 2 hours gradient of solvent A (96.9% water, 3% DMSO, 0.1 % formic acid) and solvent B (99.9% acetonitrile and 0.1% µl formic acid) in the following sequence: from 0 to 4% B in 1 min, from 4% to 30% in 79 min, from 30 to 45% B in 10 min, from 45 to 90% B in 10 min, 10 min at 90% B, from 90 to 0% B in 0.1 min, and 9.9 min at 0% B. The nanoUPLC system was coupled online to an LTQ-Orbitrap XL mass spectrometer (Thermo Scientific, Bremen, Germany). The mass spectrometer was operated in data-dependent mode to automatically measure MS1 and MS2. Data were acquired by scan cycles of one FTMS scan with a resolution of 60 000 at m/z 400

and a range from 300 to 2 000 m/z in parallel with six MS/MS scans in the linear ion trap of the most abundant precursor ions.

The mgf-files generated by Xcalibur software (Thermo Scientific, Bremen, Germany) were used for database searches with the MASCOT search engine (version 2.4.1, Matrix Science, London, UK) against the SwissProt database (SwissProt 2015_08, 549 008 sequences; 195 692 017 residues) with taxonomy human (20,278 sequences). Peptide mass tolerance for database searches was set to 7 ppm and fragment mass tolerance to 0.4 Da. Carbamidomethylation of C was set as fixed modification. Variable modifications included oxidation of M and deamidation of NQ. One missed cleavage site in case of incomplete trypsin hydrolysis was allowed. Furthermore, proteins were considered as identified if more than one unique peptide had an individual ion score exceeding the MASCOT identity threshold. Identified proteins which were present in all the IP replicates and none of the controls were considered to be interaction partners.

Split NanoLuc® Luciferase system

HEK293T cells were seeded into a white 96-well plate in 90 µL DMEM media per well. After 24 hours, cells were transfected with the pLVX plasmids containing the Nano Bit® constructs of interest using Trans-IT transfection reagent (Mirus). Luminescence signal was measured 24 hours after transfection with the Mithras LB940 plate reader (Berthold Technologies), using the Nano Glo® Luciferase assay system (Promega) according to the manufacturer's protocol. For each combination, data were normalized to the negative control, which was the respective LgBiT-tagged PFKFB4 construct incubated with Halo tagged-SmBiT. For the specificity assay, C-ter[LgBit]PFKFB4 and N-ter[SmBiT]FBXO28 were used. 50 ng of each of the constructs, along with 100 ng of empty pLVX + pLVX-PFKFB4 using varying amounts of pLVX-PFKFB4, were transfected into HEK293T cells and the assay was performed as described above.

Yeast Two Hybrid

Gateway compatible vectors for Split-Ubiquitin (pMet, pNuI and pCKZ) were a kind gift from Laurent Deslandes and Imre E. Somssich. The cDNAs in pDONR-vectors of PKFBF4 and FBXO28 were obtained from the clone repository of the DKFZ Genomics and Proteomics Core Facility. After sequence verification they were cloned into the different destination vectors using the LR-reaction protocol (Invitrogen). For all yeast work the Saccharomyces cerevisiae strain JD53 was used. The bait vector pMet and the prey vectors pNuI/CKZ were co-transformed into JD53. After transformation, yeast cells were streaked onto synthetic drop-out (SD) medium lacking histidine and tryptophan. A dilution series of the yeast strains starting with an OD600 of 1 (approximately 105 cells) were plated onto SD plates as a growth control (control), onto SD plates without uracil supplemented and with 100 mM copper sulfate (SD), and onto SD supplemented with 1 mg/mL 5-FOA and 100 mM copper sulfate (FOA). Yeast cells were grown at 30°C for 3 days and colonies were documented with a normal office scanner. Colonies on FOA plates indicate interaction of the two proteins in this yeast.

Western Blot Analysis

Prostate and lung carcinoma and control tissue lysates were obtained from Protein Biotechnologies. For protein extraction from cells, cells were washed twice with PBS and resuspended in RIPA-lysis buffer (Sigma-Aldrich; R0278) supplemented with 10 mM NaF, 10 mM Na_3VO_4 and Complete Mini Protease Inhibitor Cocktail (Roche; 11836170001). The lysate was added to QIAShredder™ Columns (Qiagen;12566) and centrifuged for 2 minutes at 13,000 rpm. Cell lysates were quantified by BCA assay, separated in 4-12 % polyacrylamide precast gels (Life Technologies; NP0321BOX) under reducing conditions and transferred onto a PVDF membrane. Immunoreactive bands were detected using ECL or ECL Plus Substrate (Thermo Scientific; PIER32106/32132). Band intensity was quantified using Image J software (v1.45s). For immunodetection of HIF-1α, cells were lysed in 1 % SDS/PBS supplemented with 10 mM NaF, 10 mM Na_3VO_4 and Complete Mini Protease Inhibitor Cocktail (Roche) and subjected to three freeze-thaw cycles in liquid nitrogen. Proteins were precipitated using 1 volume methanol and ¼ volume of chloroform and resuspended in 5 % SDS. Primary

antibodies against PFKFB4 (monoclonal, produced in-house), PDK1 (Cell Signaling; 3062S), PDH (Santa Cruz; sc-377092), p-PDH (Millipore, ABS204), Ubiquitin (Sigma; U-0508), HA (Cell Signaling, 3724), CA9 (proteintech; 11443-1-AP), SKP1 (NEB; 12248S) and CUL1 (Santa Cruz; sc-17775) were used at a 1:1 000 dilution; anti-β-actin (Cell Signaling; 3700S) was used at a 1:5 000 dilution and anti-HIF-1α (BD Biosciences; 610958), and anti-FBXO28 (Santa Cruz; sc-376851) were used at a 1:500 dilution. Horseradish peroxidase (HRP) coupled secondary antibodies (Cell Signaling; mouse 7076S, rabbit 7074S) were used at dilutions of 1:5 000.

Supplementary References

1. Campeau E, Ruhl VE, Rodier F, Smith CL, Rahmberg BL, Fuss JO, et al. A versatile viral system for expression and depletion of proteins in mammalian cells. PLoS One. 2009;4(8):e6529.
2. Sanjana NE, Shalem O, Zhang F. Improved vectors and genome-wide libraries for CRISPR screening. Nat Methods. 2014;11(8):783-4.
3. Wang T, Wei JJ, Sabatini DM, Lander ES. Genetic Screens in Human Cells Using the CRISPR-Cas9 System. Science. 2014;343(6166):80-4.
4. Kondo K, Klco J, Nakamura E, Lechpammer M, Kaelin WG. Inhibition of HIF is necessary for tumor suppression by the von Hippel-Lindau protein. Cancer Cell. 2002;1(3):237-46.
5. Kohler G, Milstein C. Continuous Cultures of Fused Cells Secreting Antibody of Predefined Specificity. Nature. 1975;256(5517):495-7.
