# Supplementary material for: PFKFB4 interacts with FBXO28 to promote HIF-1α signaling in glioblastoma
Source: Oncogenesis. 2022 Sep 17;11(1):57. doi: 10.1038/s41389-022-00433-3 (PMC9482633; doi:10.1038/s41389-022-00433-3)
Supplement: Supplementary file 2 — Supplementary Figures [file 41389_2022_433_MOESM2_ESM.pdf]

PFKFB4 interacts with FBXO28 to promote HIF-1α signaling in glioblastoma

Emma Phillips[1], Jörg Balss[1], Frederic Bethke[1], Stefan Pusch[2,3], Stefan Christen[4,5], Thomas Hielscher[6], Martina Schnölzer[7], Michael N. C. Fletcher[8], Antje Habel[2], Claudia Tessmer[9], Lisa-Marie Brenner[1], Mona Göttmann[1], David Capper[2,3,10], Christel Herold-Mende[11], Andreas von Deimling[2,3], Sarah-Maria Fendt[4,5], and Violaine Goidts[1]

Supplementary Figures

Figure S1

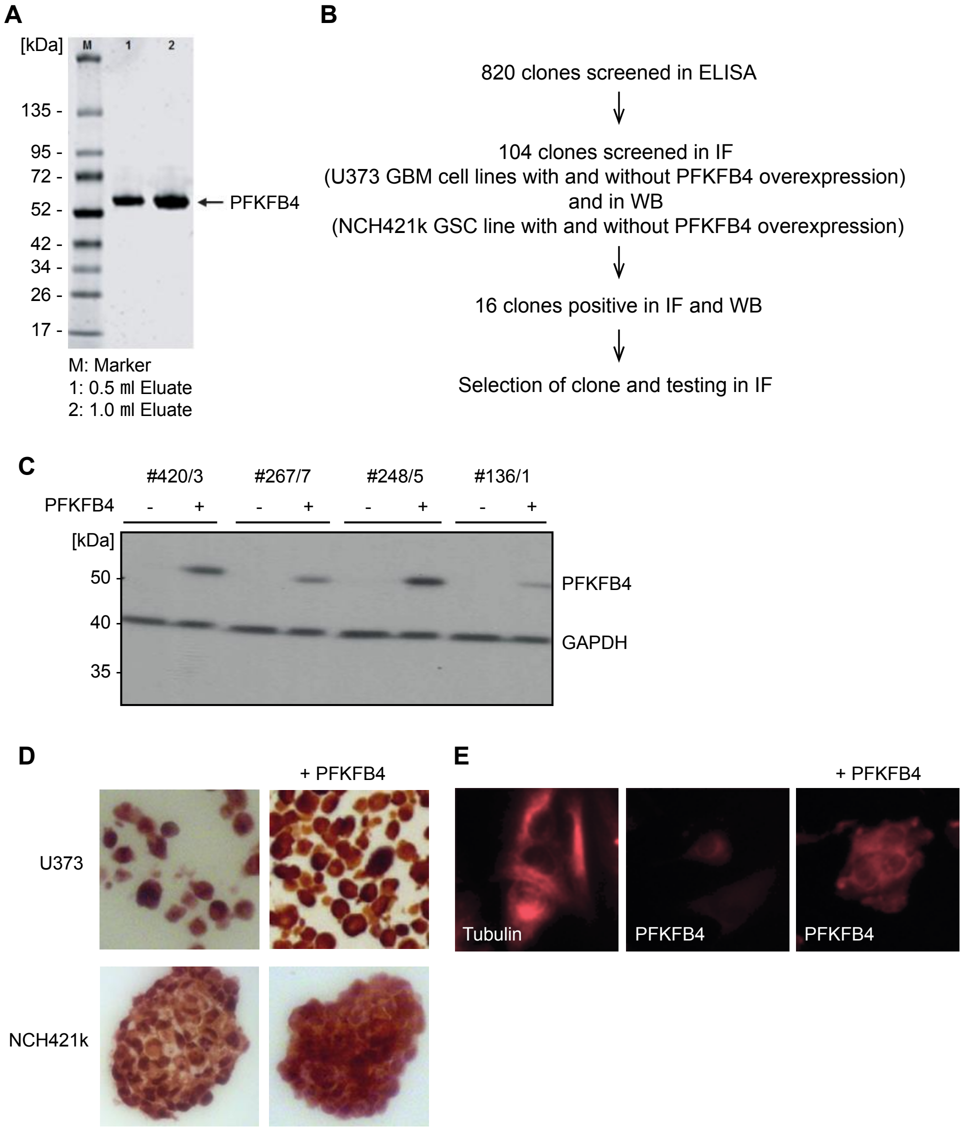

Supplementary Figure S1: Development of an anti-PFKFB4 antibody

(A) Coomassie Gel of purified PFKFB4 (purity > 98%). **(B)** Flowchart of the procedure for the selection of positive clones. **(C)** Western blot showing PFKFB4 levels in HEK293T cells with or without PFKFB4 overexpression, detected using supernatants from four different clones producing PFKFB4 antibody. GAPDH is displayed as a loading control. Clone #248/5 was used for the final antibody. **(D)** Immunohistochemical staining of PFKFB4 using supernatant from clone #248/5 in paraffin embedded U373 and NCH421k cells with or without PFKFB4 overexpression. **(E)** Immunofluorescent staining of PFKFB4 using supernatant from clone #248/5 in U373 cells with or without PFKFB4 overexpression. Read-outs were performed four days after transfection or transduction.

Figure S2

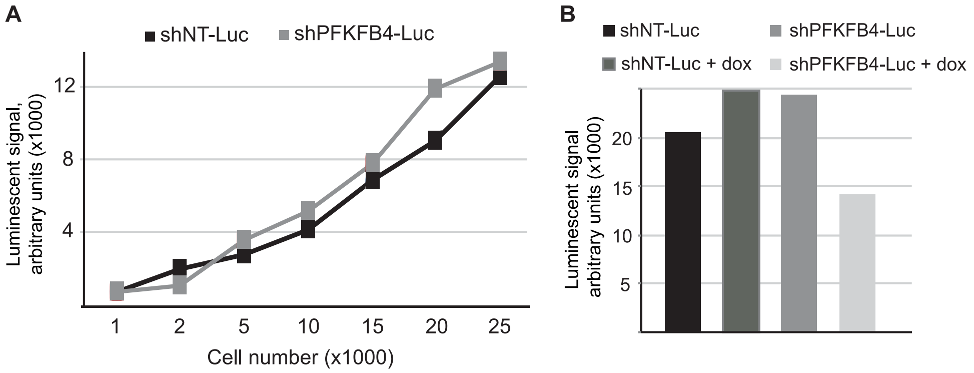

Supplementary Figure S2: Development of an *in vivo* tumor growth read-out system

(A) Bioluminescence of the doxycycline inducible shNT and shPFKFB4 luciferase expressing NCH421k GSCs seeded at densities varying from 1 000 – 25 000 cells per well. **(B)** Bioluminescence of the luciferase expressing NCH421k GSCs stably transduced with doxycycline inducible shNT or shPFKFB4 6 days with or without addition of doxycycline (n=1).

Figure S3

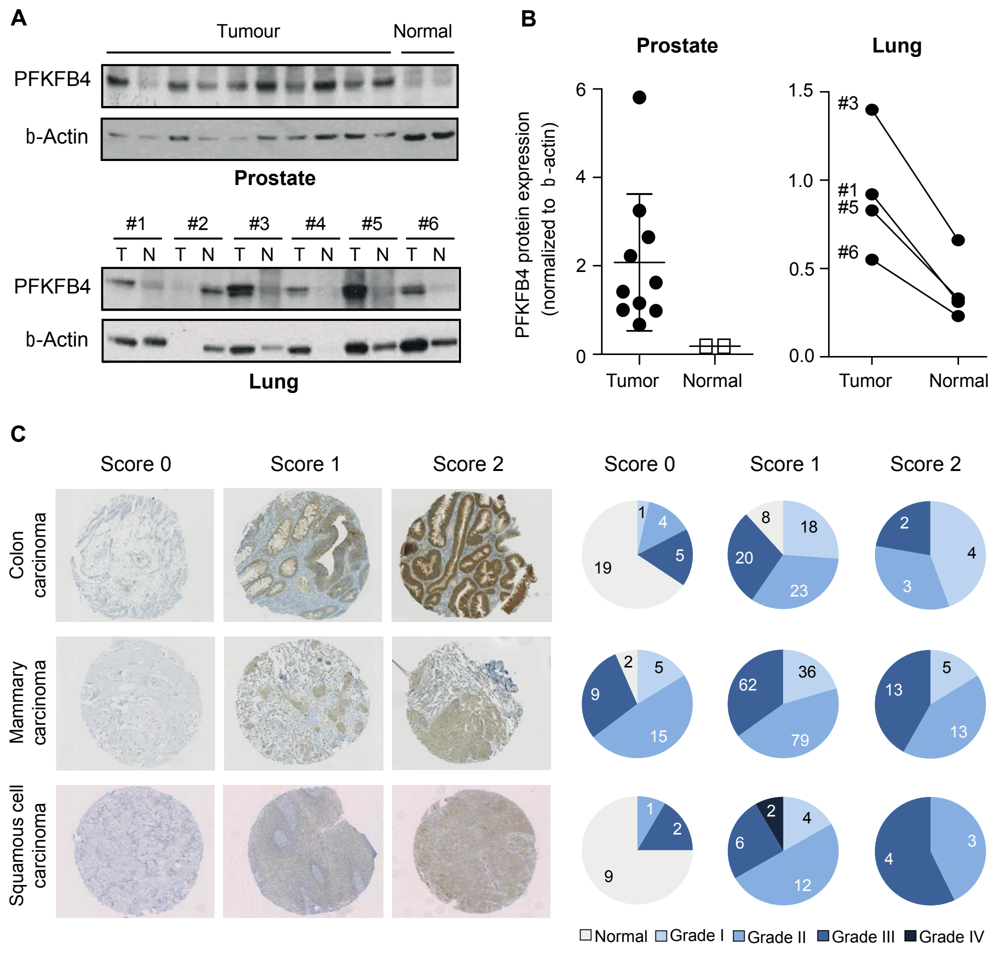

Supplementary Figure S3: Expression of PFKFB4 is Tumor Specific

(A) Protein levels of PFKFB4 from commercially available prostate carcinoma tumor lysates and normal prostate tissue lysates (upper panel) and commercially available lung carcinoma tumor lysates with matched normal controls (lower panel). β-actin is shown as a loading control. **(B)** Quantification of the prostate cancer (left) and lung cancer (right) Western blots, normalized to β-actin. For the lung cancer tumors, the tumor is paired with the matched control. Patients #2 and #4 were excluded, as no actin was detectable for one of the samples in each case. Data are represented as mean ± SD (left panel). **(C)** Left panel: representative images of PFKFB4 expression in colon, mammary and squamous cell carcinoma tumor microarrays. PFKFB4 staining was scored 0 (no expression), 1 (low expression) and 2 (high expression). Right panel: Quantification of the tumor microarrays according to scoring of PFKFB4 expression and tumor grade. The numbers in each segment denote the number of patients.

Figure S4

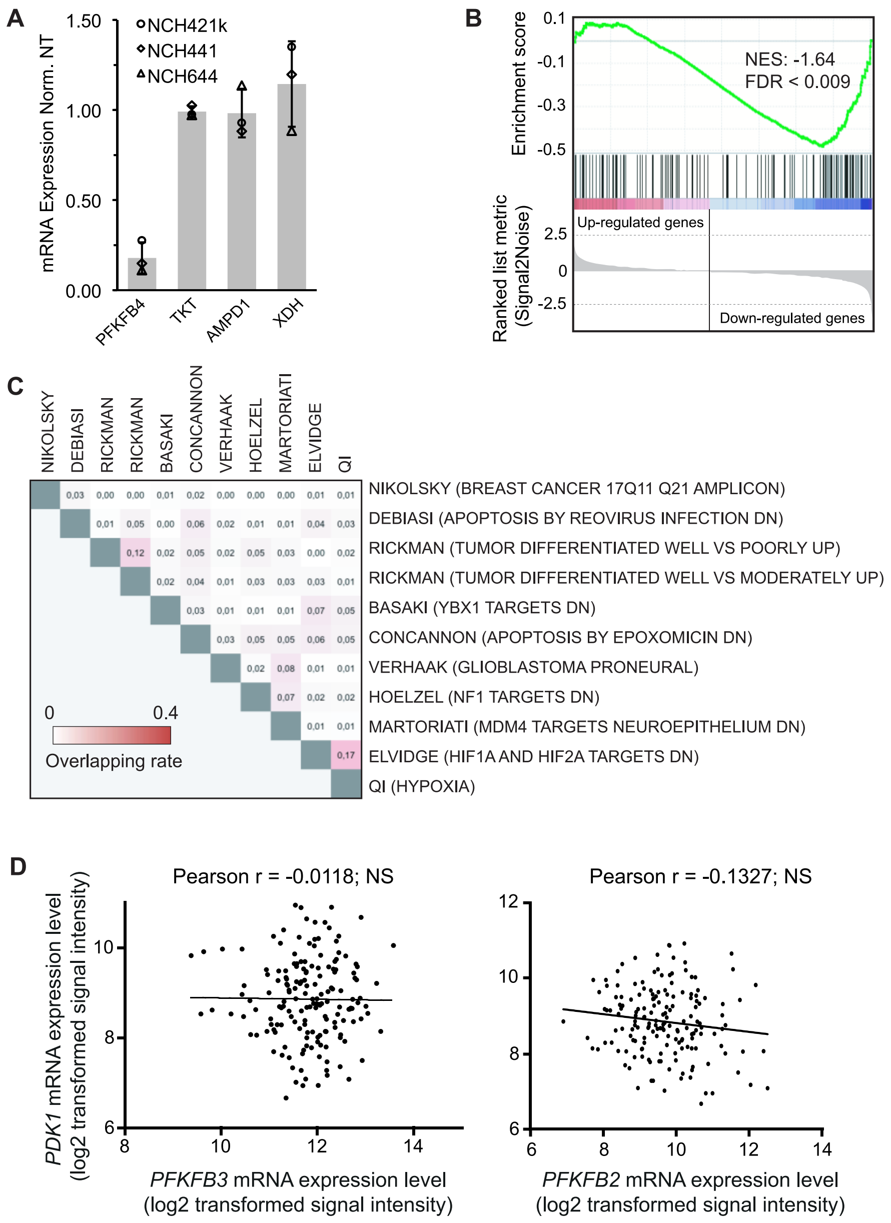

Supplementary Figure S4: Link between PFKFB4 and HIF-1α signaling

(A) mRNA levels of *PFKFB4* and the SRC-3 target genes *TKT*, *AMPD1* and *XDH* upon *PFKFB4* silencing in 3 different GSC lines, determined by gene expression profiling. **(B)** Gene Set Enrichment Analysis (GSEA) comparing a signature of genes upregulated by hypoxia in prostate cancer cells (from [28], Qi) to the gene expression list of *PFKFB4*-silenced (3 days) GSCs in a rank order based on the mean linear fold change of the genes. The green curve corresponds to the running sum of enrichment score which reflects the degree to which the hypoxia signature is overrepresented at the bottom of the list. (Normalized enrichment score (NES) = -1.64, false discovery rate (FDR) = 0.0). **(C)** Heatmap showing the overlapping rate of genes in each of the top 10 signatures shown to be downregulated upon *PFKFB4* silencing. **(D)** Correlation between *PFKFB3* or *PFKBF2* and *PDK1* mRNA expression from TCGA glioblastoma patients (regression analysis, r = -0.0118 and -0.1327, respectively, NS = not significant).

Figure S5

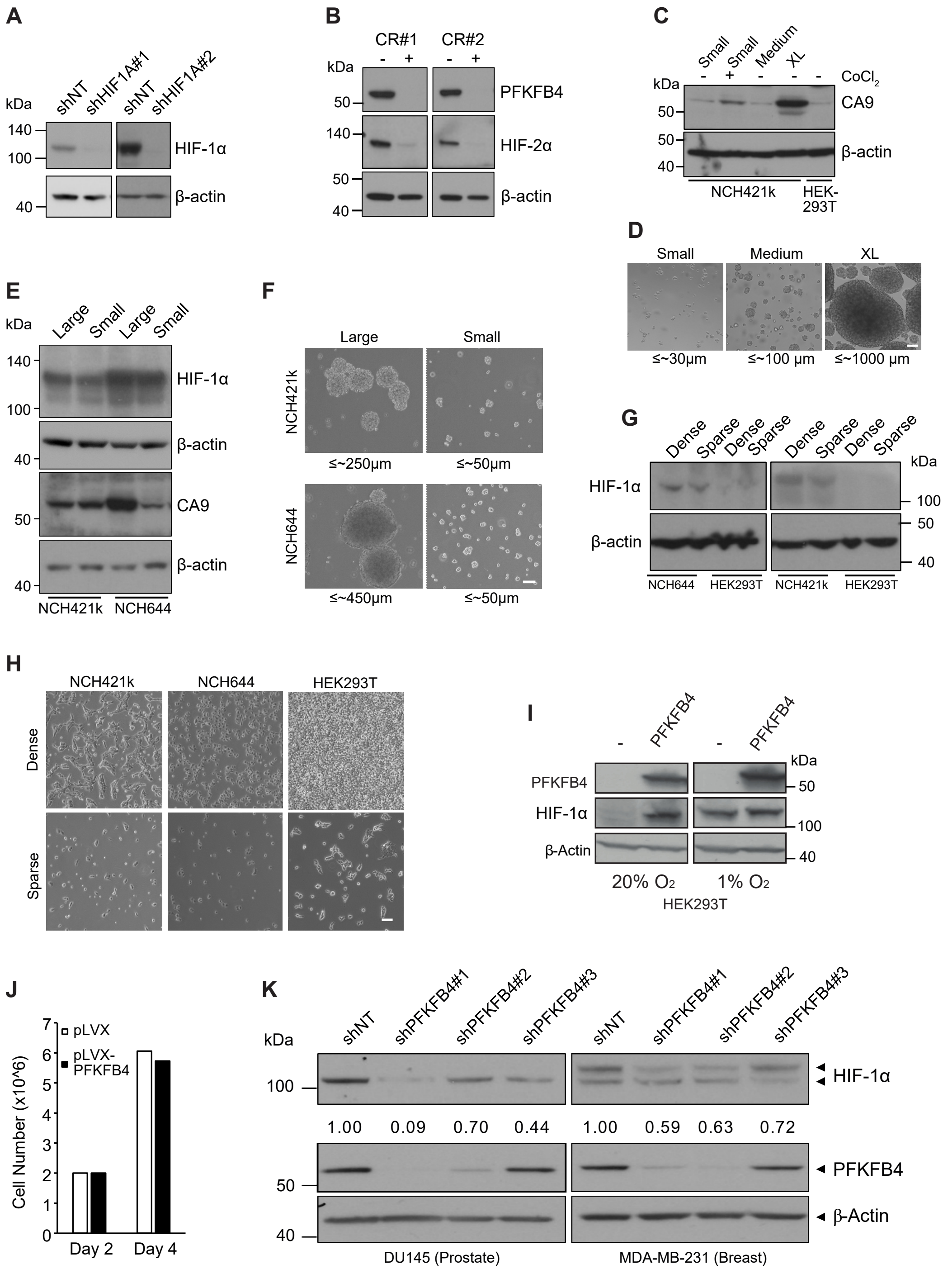

Supplementary Figure S5: HIF expression and depletion in various cellular contexts

(A) HIF-1α protein levels upon knockdown with two different shRNAs. β-actin is shown as a loading control. **(B)** Protein levels of HIF-2α after doxycycline inducible knockout of PFKFB4 (7 days incubation with dox). β-actin was used as a loading control. **(C)** CA9 levels in small (≤~30 μm), medium (≤~100 μm) and extra-large (≤~1000 μm) NCH421k cells, with or without cobalt chloride ($CoCl_2$), and HEK293T cells. β-actin was used as a loading control. **(D)** Representative images of the spheroids used in (C). Scale bar = 100 μm. **(E)** HIF-1α protein and CA9 levels in small (≤~50 μm) and large (≥250 μm) NCH421k and NCH644 spheroids. β-actin was used as a loading control. **(F)** Representative images of the spheroids used in (E). Scale bar = 100 μm. **(G)** HIF-1α protein levels in densely and sparsely growing GSCs and

HEK293T cells. β-actin was used as a loading control. **(H)** Representative images of the adherent cells used in (G). Scale bar = 100 µm. **(I)** PFKFB4 and HIF-1α protein levels in HEK293T cells in normoxia (21% O2) and hypoxia (1% O2) with and without overexpression of PFKFB4. β-actin is displayed as a loading control. **(J)** Number of HEK293T cells 2 and 4 days after transduction with empty pLVX or pLVX-PFKFB4. **(K)** Protein levels of PFKFB4 and HIF-1α upon silencing (3 days) of *PFKFB4* using three different shRNAs in DU145 (prostate cancer) and MDA-MB-231 (breast cancer) cells cultivated under hypoxic (1% O_2) conditions. β-actin is displayed as a loading control and numbers indicate quantification of HIF-1α signal normalized to β-actin signal.

Figure S6

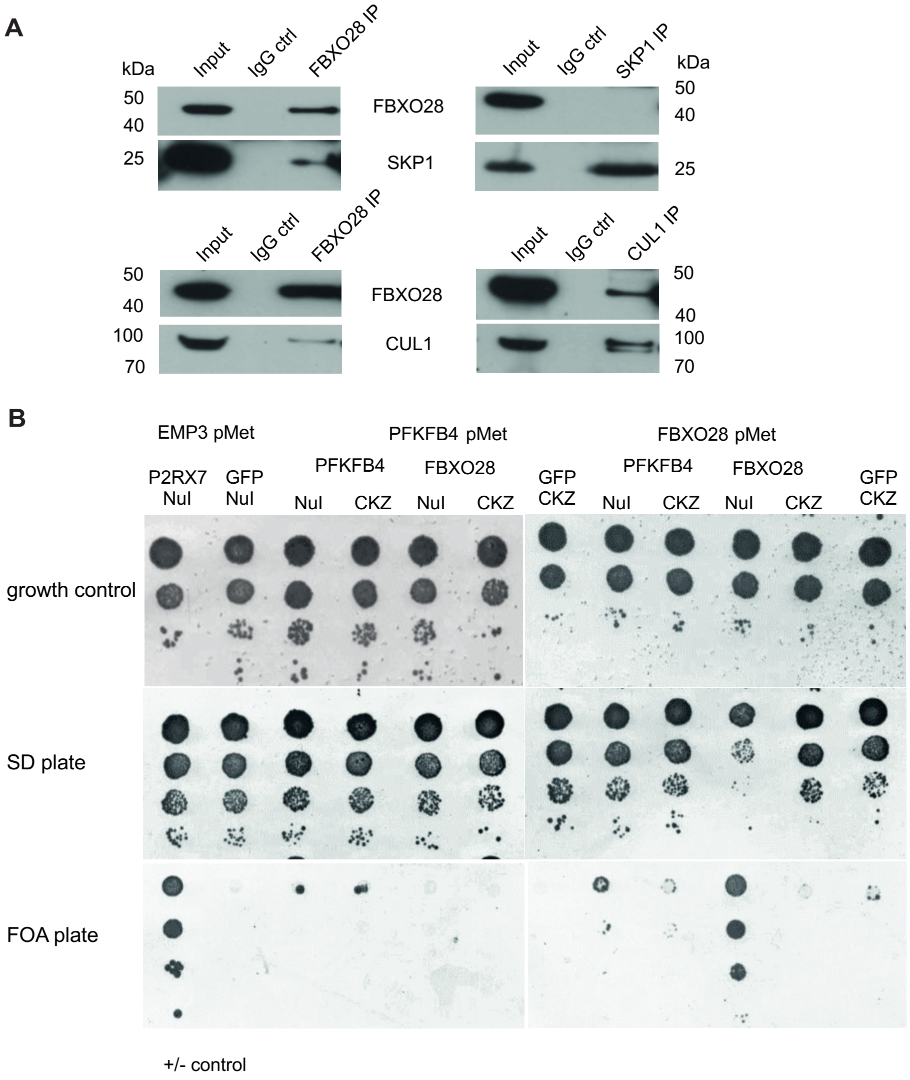

Supplementary Figure S6: PFKFB4 binds to the E3 Ubiquitin Ligase FBXO28

(A) Immunoprecipitation of FBXO28 (left, upper and lower panels), SKP1 (right, upper panel) and CUL1 (right lower panel) from HEK293T cells overexpressing FBXO28 together with SKP1 or CUL1. **(B)** Split-ubiquitin yeast two hybrid assay. Top = growth control: no selection is applied and all combinations can grow. Middle = synthetic drop-out (SD) plates: reduced growth is apparent in the case of strong interactions. Bottom = FOA plates: only yeast carrying interaction partners can grow.

Figure S7

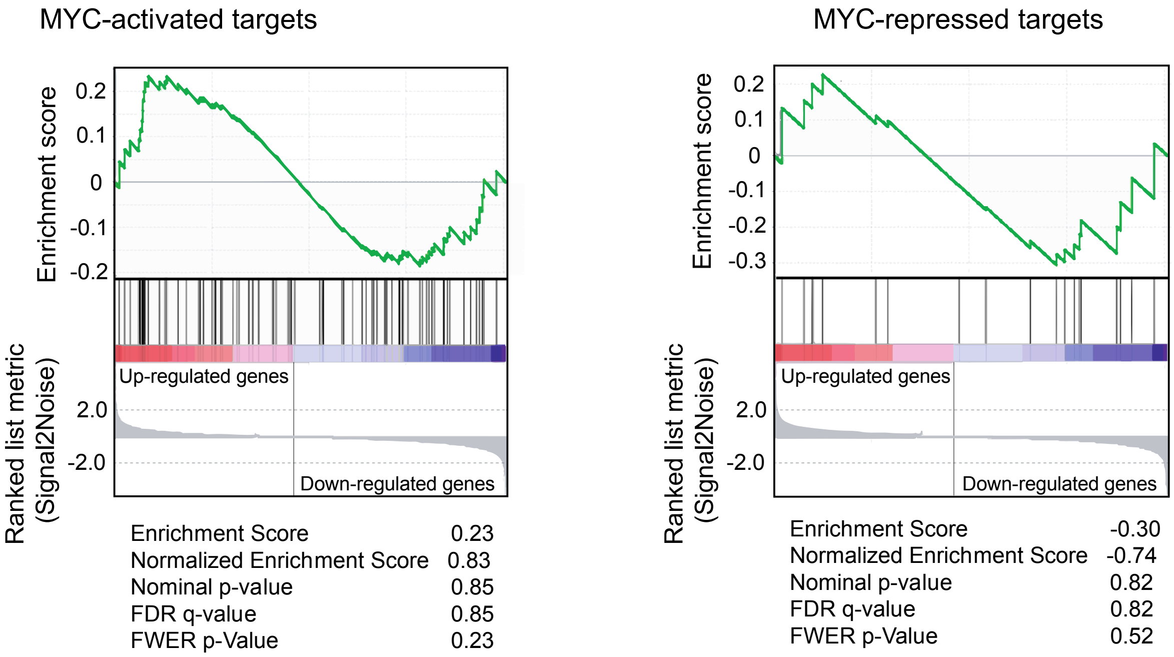

Supplementary Figure S7: *PFKFB4* silencing does not affect MYC signaling

Gene Set Enrichment Analysis (GSEA) comparing a gene signatures of MYC-activated targets (left) and MYC-repressed targets (right) to the gene expression list of *PFKFB4*-silenced GSCs in a rank order based on the mean linear fold change of the genes. The green curve corresponds to the running sum of the enrichment score which reflects the degree to which the gene signature is overrepresented at the bottom of the list. (Normalized enrichment score (NES) = 0.83 and -0.74, false discovery rate (FDR) = 0.85 and 0.82 for MYC-activated targets and MYC-repressed targets respectively).
