# Supplementary material for: PFKFB4 interacts with FBXO28 to promote HIF-1α signaling in glioblastoma
Source: Oncogenesis. 2022 Sep 17;11(1):57. doi: 10.1038/s41389-022-00433-3 (PMC9482633; doi:10.1038/s41389-022-00433-3)
Supplement: Supplementary file 3 — Supplementary Tables [file 41389_2022_433_MOESM3_ESM.pdf]

PFKFB4 interacts with FBXO28 to promote HIF-1α signaling in glioblastoma

Emma Phillips[1], Jörg Balss[1], Frederic Bethke[1], Stefan Pusch[2,3], Stefan Christen[4,5], Thomas Hielscher[6], Martina Schnölzer[7], Michael N. C. Fletcher[8], Antje Habel[2], Claudia Tessmer[9], Lisa-Marie Brenner[1], Mona Göttmann[1], David Capper[2,3,10], Christel Herold-Mende[11], Andreas von Deimling[2,3], Sarah-Maria Fendt[4,5], and Violaine Goidts[1]

Supplementary Tables

Table S1: Gene Set Enrichment Analysis of the transcriptome of GSCs upon *PFKFB4* silencing using the gene sets that represent expression signatures of genetic and chemical perturbations. Signatures with a false discovery rate of less than 25% are shown.

Rank	NAME	SIZE[a]	ES	NES	NOM p-val	FDR q-val[b]
**1**	VERHAAK GLIOBLASTOMA PRONEURAL	174	-0.573	-2.011	0	0.005
**2**	HOELZEL NF1 TARGETS DN	106	-0.602	-1.992	0	0.005
**3**	CONCANNON APOPTOSIS BY EPOXOMICIN DN	164	-0.524	-1.827	0	0.050
**4**	MARTORIATI MDM4 TARGETS NEUROEPITHELIUM DN	154	-0.500	-1.748	0	0.107
**5**	RICKMAN TUMOR DIFFERENTIATED WELL VS MODERATELY UP	102	-0.526	-1.743	0	0.109
**6**	RICKMAN TUMOR DIFFERENTIATED WELL VS POORLY UP	227	-0.484	-1.736	0	0.118
**7**	NIKOLSKY BREAST CANCER 17Q11 Q21 AMPLICON	125	-0.505	-1.724	0	0.124
**8**	BASAKI YBX1 TARGETS DN	365	-0.448	-1.678	0	0.163
**9**	ELVIDGE HIF1A AND HIF2A TARGETS DN	102	-0.511	-1.684	0	0.169
**10**	QI HYPOXIA	134	-0.484	-1.657	0.003	0.182
**11**	DEBIASI APOPTOSIS BY REOVIRUS INFECTION DN	282	-0.456	-1.659	0	0.186
**12**	NIKOLSKY BREAST CANCER 20Q12 Q13 AMPLICON	134	-0.471	-1.617	0	0.224
**13**	SENESE HDAC1 TARGETS DN	237	-0.435	-1.580	0.002	0.224
**14**	WANG CLIM2 TARGETS UP	240	-0.438	-1.578	0	0.225
**15**	HOOI ST7 TARGETS DN	114	-0.480	-1.604	0.004	0.227
**16**	ONDER CDH1 TARGETS 1 UP	136	-0.460	-1.580	0	0.228
**17**	REACTOME NEURONAL SYSTEM	275	-0.433	-1.580	0	0.229
**18**	MORI EMU MYC LYMPHOMA BY ONSET TIME UP	102	-0.480	-1.593	0.003	0.231
**19**	RODRIGUES DCC TARGETS DN	120	-0.473	-1.600	0	0.232
**20**	BLUM RESPONSE TO SALIRASIB DN	340	-0.424	-1.581	0	0.233
**21**	BERENJENO TRANSFORMED BY RHOA DN	384	-0.418	-1.566	0	0.238
**22**	XU GH1 AUTOCRINE TARGETS DN	133	-0.456	-1.553	0.001	0.239
**23**	KEGG SYSTEMIC LUPUS ERYTHEMATOSUS	124	-0.459	-1.564	0.004	0.239
**24**	REACTOME TRANSMISSION ACROSS CHEMICAL SYNAPSES	183	-0.440	-1.560	0	0.240
**25**	PECE MAMMARY STEM CELL DN	134	-0.457	-1.567	0	0.240
**26**	MARKEY RB1 CHRONIC LOF UP	112	-0.468	-1.550	0.003	0.242
**27**	NIKOLSKY BREAST CANCER 16P13 AMPLICON	108	-0.460	-1.541	0.008	0.248

[a]Gene sets of more than 100 genes were selected for the analysis. [b]The data are sorted according to the FDR.

Table S2: Interaction partners of PFKFB4 in NCH421k GSCs. PFKFB4 was immunoprecipitated from NCH421k lysate and subjected to mass spectrometry. Proteins were only considered if three or more unique peptide sequences were identified.

Gene Symbol	Mass [kDa]	Replicate	Mascot Score	# significant peptides	Coverage [%]
PFKFB4	54.7	1	1413	20	57
		2	978	15	44
		3	1210	21	56
		Ctr 1	not detected		
		Ctr 2	not detected		
PFKFB2	59	1	751	12	29
		2	686	10	30
		3	893	14	37
		Ctr 1	not detected		
		Ctr 2	not detected		
FBXO28	41.4	1	423	5	18
		2	634	10	30
		3	636	9	30
		Ctr 1	not detected		
		Ctr 2	not detected		
BDH2	27	1	350	5	30
		2	434	6	34
		3	563	8	43
		Ctr 1	not detected		
		Ctr 2	not detected		
PFKFB3	60.4	1	469	7	15
		2	349	5	15
		3	419	7	20
		Ctr 1	not detected		
		Ctr 2	not detected		

Table S3: Information about patient-derived glioblastoma stem-like cell lines (PFS = progression free survival, OS = overall survival).

Cell Line	Sex	Age (Years)	PFS (months)	OS (Months)
NCH421k	M	66	15	34
NCH441	M	75	22	31
NCH644	F	67	17	30

Table S4: shRNA nucleotide sequences

shRNA	Nucleotide Sequence
shNT	CAACAAGATGAAGAGCACCAA
PFKFB4-sh1	CCTGTGGCATATGGTTGTAAA
PFKFB4-sh2	GACGTGGTCAAGACCTACAAA
PFKFB4-sh3	GCTGATTGGCTGCCACATTTC
FBXO28	GAGAATGTTGAATCAGGGATT
*HIF1A*_UTR	TGCTCTTTGTGGTTGGATCTA
HIF1A	CCGCTGGAGACACAATCATAT

Table S5: sgRNA sequences

sgRNA	Nucleotide Sequence
PFKFB4 CRISPR_1	TGTAGGTCTTGACCACGTCC
PFKFB4 CRISPR_2	TGACCACGTCCCGGCGATAC
PFKFB3 CRISPR_1	CAACGTCGGGGAGTATCGCC
PFKFB3 CRISPR_2	TCAACGTCGGGGAGTATCGC
PFKFB3 CRISPR_3	CTTCTTCCGCCCCGACAATG

Table S6: Cloning primers

Primer	Nucleotide Sequence (5'-3')
FBXO28_F	GATCgaattcATGGCGGCAGCGGCG
FBXO28_R	GCGCGtctagaGGGACACCATGTTGC
PFKFB4_F	ACTGgaattcATGGCGTCCCCACGG
PFKFB4_R	ACTGtctagaTCACTGGTGAGCAGGCACC
SKP1_F	GGCCggatccATGCCTTCAATTAAGTTGCAGAG
SKP1_R	GCGCaccgttTCACTTCTCTTCACACCACTG
CUL1_F	GGCCggatccATGTCGTCAACCCGGAGC
CUL1_R	GCGCaccgttTTAAGCCAAGTAACTGTAGGTGTCC
HIF1A_F	GCTAgggcccTACCATGGCCTACCCCTA
HIF1A_R	CGATtctagaGCCGCCAGTGTGATGGATA
NanoBiT® PFKFB4 C-term_F	ACTGacgctaGCACCATGGCGTCCCCACGG
NanoBiT® PFKFB4 C-term_R	ACTGacgaatTCCCCTGGTGAGCAGGCACC
NanoBiT® FBXO28 C-term_F	ACTGacgctaGCACCATGGCGGCAGCGGCG
NanoBiT® FBXO28 C-term_R	ACTGacgaatTCCCCTTTCTATTCCGAAGACG
NanoBiT® PFKFB4 N-term_F	ACTGacgaatTCAGCGTCCCCACGGGAA
NanoBiT® PFKFB4 N-term_R	ACTGactctaGACTGGTGAGCAGGCACC
NanoBiT® FBXO28 N-term_F	ACTGacgaatTCAGCGGCAGCGGCGGAG
NanoBiT® FBXO28 N-term_R	ACTGactctaGATTACTTTCTATTCCGAAGACG
N-LgBiT_F	ACTGacttcgAAATGGTCTTCACACTCG
N-SmBiT_F	ACTGacttcgAAATGGTGACCGGCTACC
C-LgBiT _R	ACTGactctaGATTAGCTGTTGATGGTTACTCGG
C-SmBiT _R	ACTGactctaGACTAGATTACAGAATCTCCTCG
PFKFB4 _F	ACTGacttcgAAATGGCGTCCCCAC
FBXO28 _F	ACTGacttcgAAATGGCGGCAGCGG
HaloTag _F	ACTGacttcgAAATGGCAGAAATCGGTACTGG
HaloTag _R	ACTGactctaGACGGCCTTACAGAATCTCC

Table S7: Mutagenesis primers

Primer	Nucleotide Sequence (5'-3')
Mut_Hif1a_A1595G_F	AATGAATTCAgGTTGGAATTGG
Mut_Hif1a_A1595G_R	GACCATATCACTATCCAC
Mut_Hif1a_A1613G_A1640G_F	ACAGAAGCAAgGAACCCATTTTC
Mut_Hif1a_ A1613G_A1640G_F	GTCTTCAGCAAAAAGTcTTTC

Table S8: RT-PCR primers

Primer	Nucleotide Sequence
ARF1 fw	GACCACGATCCTCTACAAGC
ARF1 rev	TCCCACACAGTGAAGCTGATG
DCTN2 fw	CGCCATGGCGACCCTAAAT
DCTN2 rev	TTGTCAGCTCCTCCGCATCGAA
HIF1A fw	AACCTGATGCTTTAACTTGCTG
HIF1A rev	CTTCCTCAAGTTGCTGGTCA
HPRT fw	TGACACTGGCAAAACAATGCA
HPRT rev	GGTCCTTTTCACCAGCAAGCT
PFKFB4 fw	CCACTCGGGAGTTCAATGTT
PFKFB4 rev	TTTTCAGGCCCTCTTCATTG
